# MJDs family members: Potential prognostic targets and immune-associated biomarkers in hepatocellular carcinoma

**DOI:** 10.3389/fgene.2022.965805

**Published:** 2022-09-09

**Authors:** Lei Zhou, Guojie Chen, Tao Liu, Xinyuan Liu, Chengxiao Yang, Jianxin Jiang

**Affiliations:** ^1^ Department of Hepatobiliary Surgery, The Affiliated Hospital of Guizhou Medical University, Guiyang, China; ^2^ School of Clinical Medicine, Guizhou Medical University, Guiyang, China; ^3^ Hunan YoBon Biotechnology Limited Company, Changsha, China; ^4^ Department of Hepatobiliary Surgery, Renmin Hospital of Wuhan University, Wuhan, China

**Keywords:** MJDs family members, hepatocellular carcinoma, methylation, immune cell infiltration, prognostic biomarker

## Abstract

Hepatocellular carcinoma (HCC) is one of the most common gastrointestinal malignancies. It is not easy to be diagnosed in the early stage and is prone to relapse, with a very poor prognosis. And immune cell infiltration and tumor microenvironment play important roles in predicting therapeutic response and prognosis of HCC. Machado-Joseph domain-containing proteases (MJDs), as a gene family extensively involved in tumor progression, has pro-cancer and anti-cancer effects. However, the relationship between MJDs family members and immune cell infiltration and tumor microenvironment in HCC remains unclear. Therefore, cBio Cancer Genomics Portal (cBioPortal), The Cancer Genome Atlas (TCGA), UALCAN, Human Protein Atlas (HPA), MethSurv, and Tumor Immune Estimation Resource (TIMER) databases were performed to investigate the mRNA expression, DNA methylation, clinicopathologic features, immune cell infiltration and other related functions of MJDs family members in HCC. The results indicated that the expression of ATXN3, JOSD1, and JOSD2 was dramatically increased in HCC tissues and cell lines, and was correlated with histological grade, specimen type, TP53 mutation, lymph node metastatic, gender, and age of patients with HCC. Meanwhile, these genes also showed clinical value in improving the overall survival (OS), disease-specific survival (DSS**)**, progression free survival (PFS), and relapse-free survival (RFS) in patients with HCC. The prognostic model indicated that the worse survival was associated with overall high expression of MJDs members. Next, the results suggested that promotor methylation levels of the MJDs family were closely related to these family mRNA expression levels, clinicopathologic features, and prognostic values in HCC. Moreover, the MJDs family were significantly correlated with CD4^+^ T cells, CD8^+^ T cells, B cells, neutrophils, macrophages, and DCs. And MJDs family members’ expression were substantially associated with the levels of several lymphocytes, immunomoinhibitors, immunomostimulators, chemokine ligands, and chemokine receptors. In addition, the expression levels of MJDs family were significantly correlated with cancer-related signaling pathways. Taken together, our results indicated that the aberrant expression of MJDs family in HCC played a critical role in clinical feature, prognosis, tumor microenvironment, immune-related molecules, mutation, gene copy number, and promoter methylation level. And MJDs family may be effective immunotherapeutic targets for patients with HCC and have the potential to be prognostic biomarkers.

## Introduction

Hepatocellular carcinoma (HCC) is the fifth most common cancer, and is also the second causing reason for cancer-related deaths. According to National Cancer Center (NCC), HCC is the first cause of death for men aged 15–59 years in China (Zheng et al., 2022). In recent years, with the continuous improvement of diagnostic techniques and systemic treatment, the prognosis of HCC patients has been greatly improved. Unfortunately, the 5-year survival rate of HCC is still less than 19% (Feng et al., 2020). Thus, we need to further explore markers for early diagnosis and related therapeutic targets of HCC to improve the prognosis of patients.

The MJDs family, the sub-classes of deubiquitinases, consists of four members: ATXN3, ATXN3L, JOSD1, and JOSD2. The MJDs family members is of critical importance in tumorigenesis and tumor progression (Zeng et al., 2020). For example, downregulation of ATXN3 promotes sensitivity of neuroblastoma cells to Perifosine and MK-2206, but reduces sensitivity to etoposide and cisplatin in these cells (Gong et al., 2021); ATXN3 enhances breast cancer (BRCA) metastasis by deubiquitinating KLF4 (Zou et al., 2019); ATXN3 promotes mRNA expression of EIF5A2 by stabilizing EIF5A2 to decrease its ubiquitination and degradation in anaplastic thyroid carcinoma (Zhuang et al., 2021). Down-regulation of ATXN3L suppresses breast cancer cell proliferation by directly binding to KLF5 (Ge et al., 2015). Meanwhile, ATXN3L is also presented to promote the migration of NSCLC cells (Buus et al., 2009). JOSD1 suppresses mitochondrial apoptosis to induce the chemotherapy drug resistance in the gynaecological tumor by deubiquitinating and stabilizing MCL1 (Wu et al., 2020). In acute myeloid leukemia (AML) cells, JOSD1 interacts with JAK2-V617F and promotes its expression (Yang et al., 2022). JOSD2 deficiency inhibits tumor cell proliferation by reducing glycolysis, and its mRNA expression is related to the worst prognosis in NSCLC (Krassikova et al., 2021). JOSD2 directly binds to and reduces ubiquitination levels of CTNNB1, thus, augmenting Wnt pathway transduction in HCC. In addition, Over-expression of JOSD2 is positively associated with poor prognosis in HCC patients (Huang et al., 2022). However, the possible biological functions of MJDs family members in HCC and its prognostic value remain unclear.

In our study, we analyzed the mRNA expression alterations in MJDs family members, clinical characteristics, prognostic values, promoter methylation levels, MJDs members’ gene changes, their association with immune infiltration cells, and immunomodulators. The study shows the underlying biological functions and the prognostic values of MJDs family members, which will facilitate the early diagnosis, targeted therapy, and immunotherapy of HCC patients.

## Materials and methods

### UALCAN

UALCAN is a synthetically web-based database for analyzing TCGA data. In this study, HCC sample types, ages, genders, grades, tumor stages, lymph node metastasis TP53 mutations, and promotor methylation levels were from the UALCAN database.

### GTEx

This database integrates multi-omics data based on various normal human tissues. According to the Genotype-Tissue Expression (GTEx) databases in normal tissues, the expression of MJDs family members were analyzed.

### cBioPortal

cBioPortal is an integrated web tool to analyze the TCGA database. We used this tool to analyze the gene structure variation of MJDs family members, co-expression, correlation between methylation levels and mRNA expression levels.

### MethSurv

MethSurv is a web tool to study the relationship between aggregation of methylation sites and patient survival. We used this tool to analyze the methylation levels of CpG island in members of the MJDs family and their relationship with patient survival.

### Kaplan-Meier analysis

The association between the expressed level of MJDs family members and the outcome of HCC patients was analyzed with the Kaplan-Meier. In the study, we evaluated outcomes with HCC patients through means of disease-specific survival (DSS), overall survival (OS), and progression free survival (PFS) as well as relapse-free survival (RFS) curves.

### Construction of prognostic signature model

Downloading the STAR-counts data and clinical details of patients with HCC from the TCGA database (https://portal.gdc.com), normalizing the data to log2 (TPM+1), and then keeping the data with samples of RNAseq data and clinical details allowed us to divide the patients into high-risk and low-risk groups for further analysis. The timeROC analysis was carried out to determine the accuracy of the prediction model, and the log rank was utilized to assess the KM survival analysis to compare the survival difference between the two groups mentioned above. The 10-fold cross-validation method and the least absolute shrinkage and selection operator (LASSO) regression algorithm were used to pick the features. R’s glmnet package was used for the analysis mentioned above. The log rank test and univariate Cox regression were used to provide *p*-values and hazard ratios (HR) with 95 percent confidence intervals (CI) for Kaplan-Meier curves. R software version 4.0.3 was used to carry out all of the aforementioned analysis techniques and R packages (R 4.0 Foundation for Statistical Computing, 2020). Statistics were deemed significant at p < 0.05.

### GSCALite

The GSCALite is a versatile database analysis web tool that we use to analyze the signaling pathways activated or inhibited by MJDs family members.

### TIMER immune score of MJDs

The TCGA database (https://portal.gdc.com), which contains RNAseq data, was used to gather the clinical data and related data for patients with liver cancer. Immunedeconv, a R software package that incorporates the TIMER algorithm, has been rigorously benchmarked and each approach has been proven to have its own distinct performance and advantages. Immunedeconv was used to reliably evaluate immunity scores. The ggplot2 and pheatmap R (v4.0.3) packages were used to produce the aforementioned findings.

### TISIDB

TISIDB is an integrated database for the study of tumor-immune system interactions, allowing for the analysis and annotation of 10 types of data. The correlations between MJDs family members with lymphocytes and immunomodulators in HCC were analyzed by using the TISIDB database.

### Immune checkpoints analysis

371 liver cancer patients’ RNAseq data and relative clinical data were gathered from the TCGA database (https://portal.gdc.com). Immune checkpoint genes include SIGLEC15, TIGIT, CD274, HAVCR2, PDCD1, CTLA4, LAG3, and PDCD1LG2. To track the expression of genes linked to immunological checkpoints, the expression values of these eight genes were identified. The R packages ggplot2 and pheatmap were used to produce the aforementioned findings.

### Cell culture

Normal liver cell line HL-7702 and HCC cell lines (MHCC-97H, HepG2, PLC/PRF/5, Huh7, and HCCLM-3) were provided from the Department of Biliary-Pancreatic Surgery of Cell Line Resource, Tongji Hospital (Hubei, Wuhan, China). All HCC cell lines were cultured in DMEM (Meisen, Zhejiang, China) and HL-7702 was cultured in RPMI-1640 (Meisen, Zhejiang, China). Both DMEM and RPMI-1640 were added to 10% of decomplemented fetal bovine serum (Gibco,USA) and 1% of penicillin/streptomycin (BI, Israel). Moreover, the mentioned above cells were incubated at a suitable temperature (37°C) and 5% carbon dioxide (5% CO_2_).

### RNA extraction and qRT-PCR assay

According to the protocol, total RNA was collected from HL-7702 and HCC cell lines using TRIzol reagent. The extracted total RNA was then subjected to RT-PCR using HiScript III RT SuperMix (Vazyme, Nanjing, China) and the corresponding primers to obtain complementary DNA (cDNA). Using the qPCR Master Mix (SYBR Green, Vazyme, Nanjing, China), qRT-PCR was performed in a CFX96 Real-Time PCR detection system (Bio-Rad, USA). To analyze the relative expression of genes was calculated using the 2^−ΔΔCt^ method. The primer sequences of the genes were listed in Supplementary Table S1.

### Human Protein Atlas (HPA)

The HPA database integrates transcriptomics and proteomics, which includes different profiles of normal and abnormal tissue types and various cell types. The immunohistochemical (IHC) staining images of HCC tissues and normal liver tissues of MJDs family members from the HPA database.

## Results

### Pan-cancer analysis of MJDs family members’ expression

**FIGURE 1:**
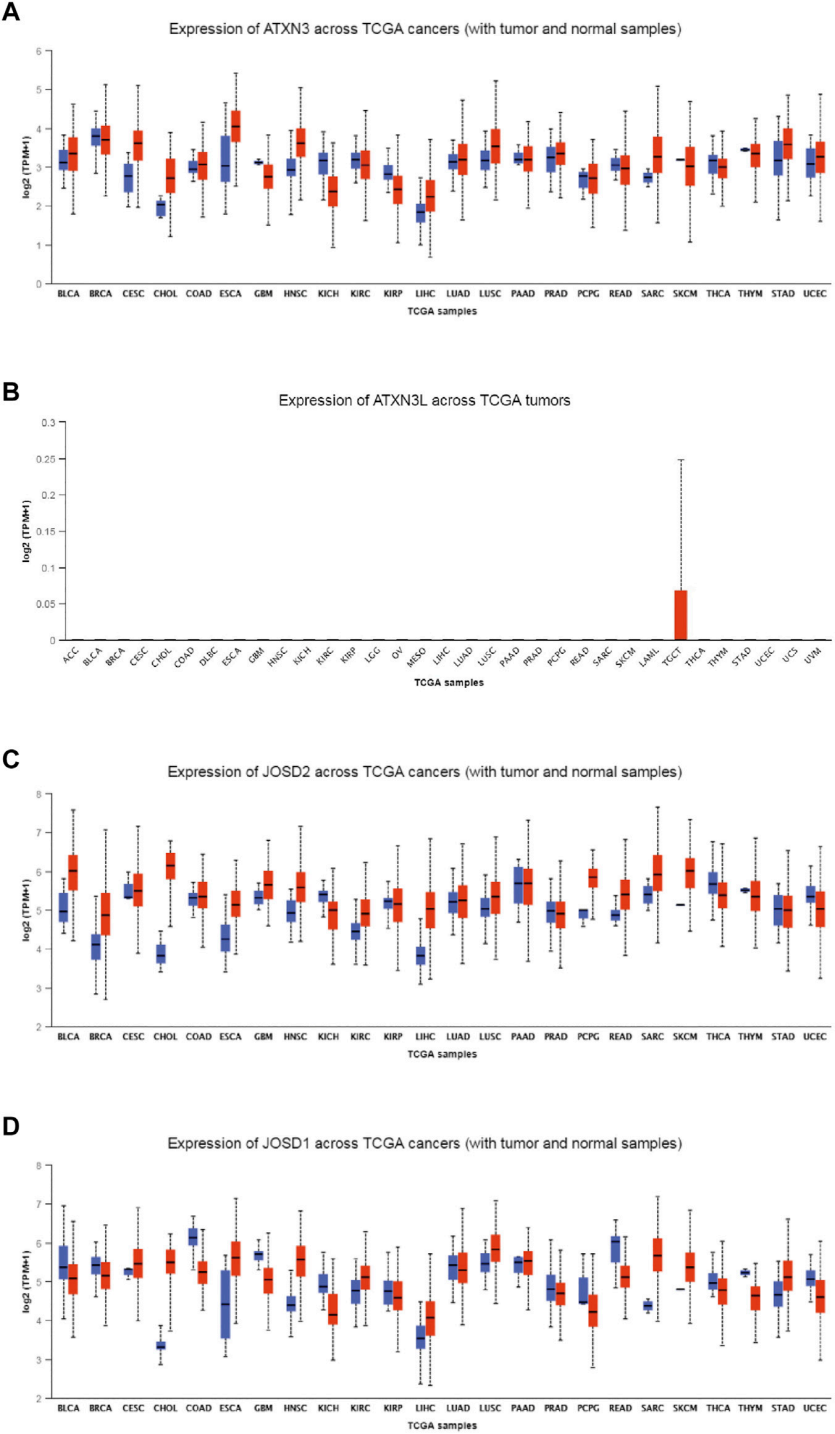
**(A–D)** The expression levels of MJDs family members in different types of cancers. The mRNA expression levels of ATXN3, ATXN3L, JOSD1, and JOSD2 were compared with various TCGA cancers.

To explore the expression levels of MJDs family members in HCC, we used the UALCAN database to find different transcriptional levels of MJDs members in pan-cancer. We identified three genes of the MJDs family with abnormal expression in HCC tissues. ATXN3, JOSD1, and JOSD2 were upregulated, while ATXN3L was only expressed in testicular tissue and testicular carcinoma tissue (Figure 1). The expressions of MJDs family members in normal tissues was then obtained from GTEx database, ATXN3L was only expressed in testicular tissue, and the other three genes were expressed in all tissues (Supplementary Figure S1).

### Correlation between MJDs family Members’ expression

Based on UALCAN database, the correlation analysis between MJDs family members’ expression and histological grades, sample types, TP53 mutations, lymph node metastatic status, genders, and years were further shown.

**FIGURE 2:**
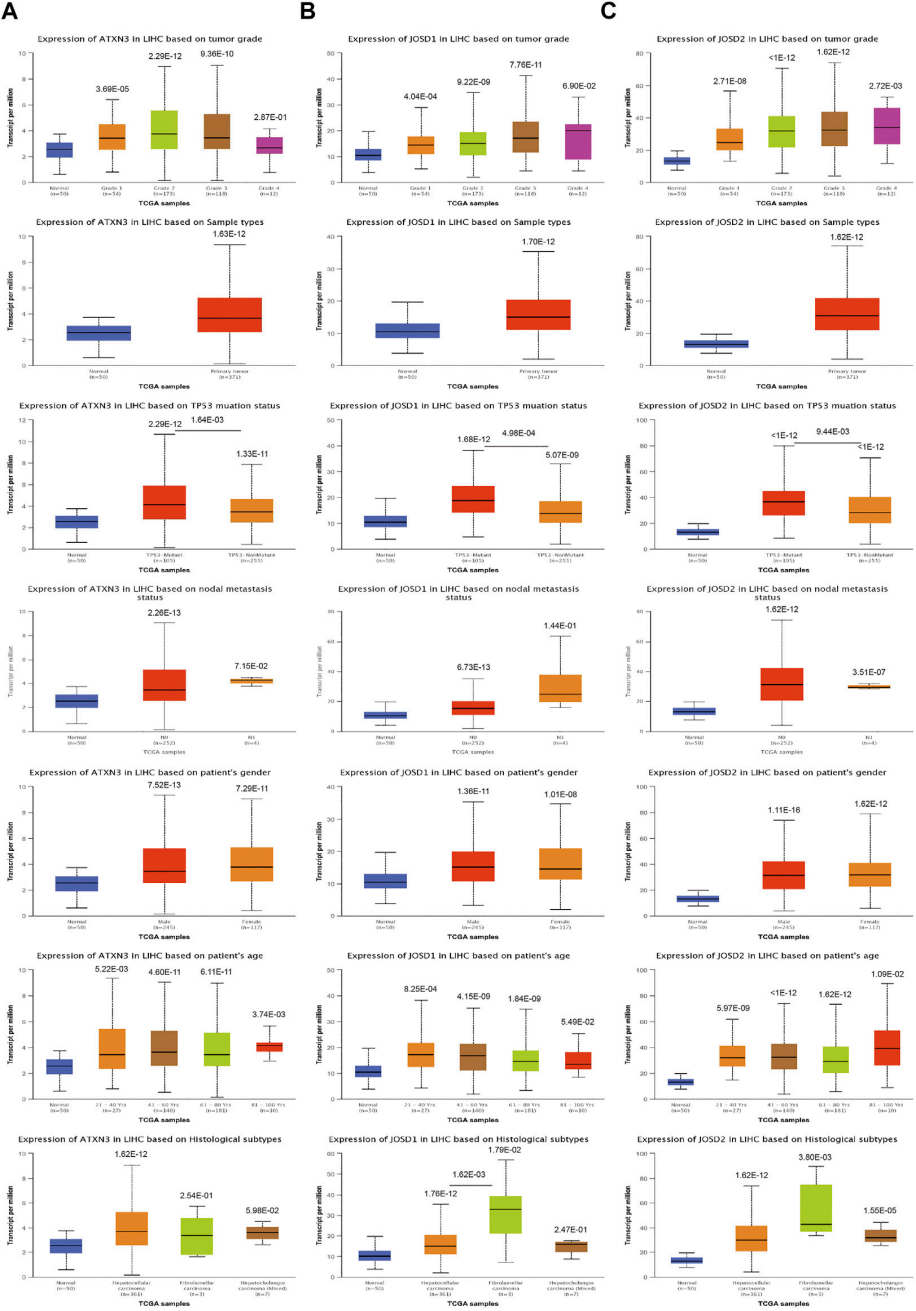
**(A–D)** The transcription level of MJDs family members in the subgroup of HCC patients, and classified by according to histological grades, sample types, TP53 mutations, lymph node metastatic status, genders, years and histological subtypes.

As shown in Figure 2A, mRNA expression levels of ATXN3 were significantly related to histological grades, sample types, TP53 mutations, lymph node metastatic status, genders, and years. Specifically, In HCC patients, the expression of ATXN3 showed an increasing tendency in stages 1–3. The expression of ATXN3 in HCC tissues was notably higher than that in normal liver tissues. HCC specimens with non-mutant TP53 and mutant TP53 had observably higher ATXN3 expression levels than the control group. In the lymph node metastasis group, the mRNA expression levels of ATXN3 in both N0 and N1 groups were observably higher than that in the normal control. The mRNA expression level of ATXN3 in N1 was higher than that in N0 without statistical difference. In gender groups, the expression level of ATXN3 in both male and female groups was higher than that in the control group, which was statistically significant. However, without statistical difference, the expression level of ATXN3 in females was higher than that in males. Furthermore, ATXN3 transcriptional levels were related to different age groups. As age increases, ATXN3 expression also enhanced differently in HCC patients. The mRNA expression of ATXN3 was significantly associated with different HCC subclasses, with hepatocellular carcinoma having the highest levels and fibrolamellar carcinoma showing the lowest levels.

Similarly, the UALCAN Database was used to explore the transcriptional levels of JOSD1 and JOSD2 in HCC. We also found that JOSD1 and JOSD2 expressions remarkably difference in varied sample types, histological grades, TP53 mutations, lymph node metastatic status, genders, and years of HCC (Figures 2B,C). Therefore, the expression of ATXN3, JOSD1, and JOSD2 can be used as potential diagnostic indicators of HCC.

### Promoter methylation level of MJDs family members in HCC

**FIGURE 3:**
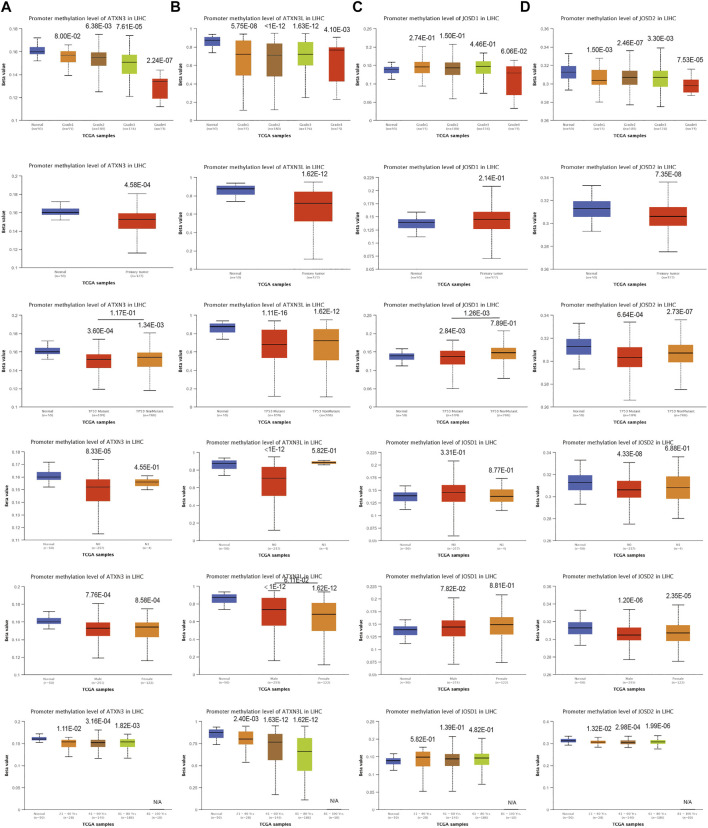
**(A–D)** Correlation analysis between methylation level of MJDs family members and HCC patients clinical characteristics. The MJDs methylation level in HCC patients classified by histological grades, sample types, TP53 mutations, lymph node metastatic status, genders, and years were shown.

To explore the mechanisms for the up-regulation of mRNA expression levels, we further used UALCAN database to analyze MJDs family members’ promoter methylation levels and their correlation with HCC patients of clinical characteristics. We found that the ATXN3 methylation β value of the HCC group and the normal group was less than 0.2, which was a completely unmethylated state. The results suggested the ATXN3 methylation levels in HCC tissues were lower than in corresponding normal groups, regardless of TP53 mutations, histological grades, lymph node metastatic, genders, and years (Figure 3A). This was the same as the up-regulated ATXN3 expression in HCC patients. In addition, we found that the ATXN3L and JOSD2 promoter methylation levels were significantly lower in HCC tissues than that in normal tissues, despite histological grades, TP53 mutations, lymph node metastatic, genders, and years (Figures 3B,D). Interestingly, we found that the methylation β value of ATXN3L in various subgroups of years, genders, histological grades, lymph node metastasis and TP53 mutation was greater than 0.6, presenting a complete methylation state. In age groups, the methylation level decreased with the increasing of age, but it was still in a complete methylation state. In tumor stages, β values of G1, G2, G3, and G4 decreased, indicating the down-regulation of methylation levels. However, JOSD1 methylation levels in HCC tissues was higher than that in corresponding normal tissues without statistic difference, in spite of histological grades, TP53 mutations, lymph node metastatic, genders, and years (Figure 3C).

Moreover, we evaluated the CpG sites of promoter methylation of MJDs family members in hepatocellular carcinoma. In the heat map of methylation levels at CpG sites, we found that ATXN3 had 12 CpG islands, among which cg12034871 and cg26081025 were completely methylated, and the rest were completely unmethylated (Supplementary Figure S2A). Meanwhile, we also found that ATXN3L had four CpG islands, with partial methylation and complete unmethylation in each sample (Supplementary Figure S2B). In addition, JOSD1 had 17 CpG islands among which cg03088955 was completely methylation (Supplementary Figure S2C). There were six CpG islands in JOSD2, and only cg18708810 and cg13521229 had complete and partial methylation cases, respectively (Supplementary Figure S2D).

**FIGURE 4:**
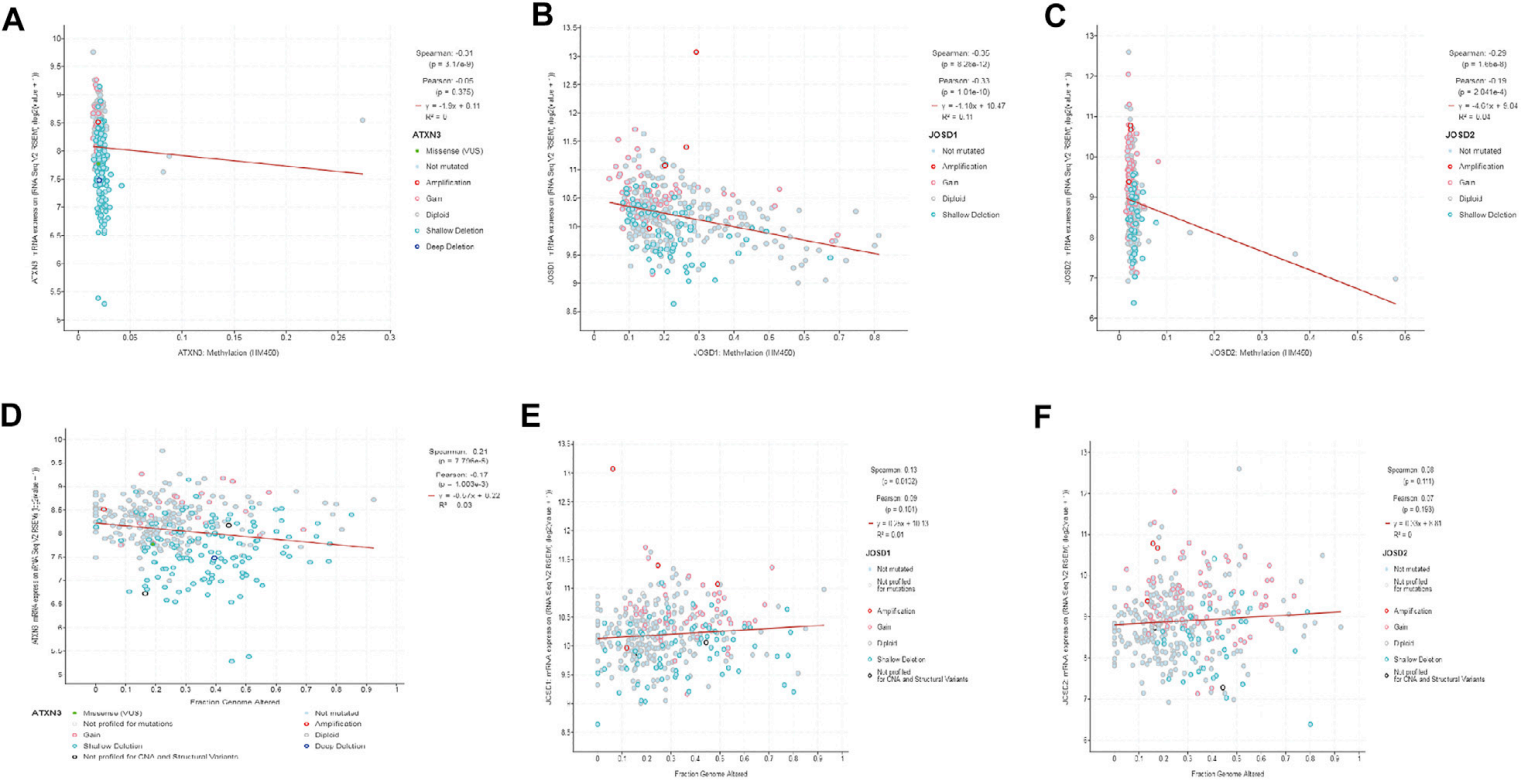
**(A–C)** The promoter methylation levels of MJDs family members were inversely correlated with the expression of MJDs family members **(D)** ATXN3 mRNA expression levels were positively associated to the fraction of the genome altered **(E,F)** The expressions of JOSD1 and JOSD2 were negatively related to the fraction of the genome altered.

Furthermore, we analyzed the correlations between the degree of methylation of ATXN3, JOSD1, and JOSD2 and the mRNA expression levels with the cBioPortal database. The results demonstrated that the Spearman coefficients of ATXN3, JOSD1, and JOSD2 were -0.31,-0.35, and -0.29, respectively, indicating negative association between the methylation levels and the mRNA transcriptional levels of these three genes in HCC patients (Figures 4A–C). Meanwhile, the correlations between the fraction genome alteration and the mRNA expression levels were analyzed, and we found that the Spearman coefficients of ATXN3 was -0.21, which was a negative correlation, while the Spearman coefficients of both JOSD1 and JOSD2 were 0.13 and 0.08, respectively, which were positive correlations (Figure 4 D, E, F). Thus, the results suggested that lower levels of ATXN3, JOSD1, and JOSD2 promoter methylation levels might lead to higher mRNA transcriptional levels of these genes in HCC. There may be other mechanisms of mRNA up-regulation of these three genes in HCC patients.

### Analysis of MJDs alterations in HCC

**FIGURE 5:**
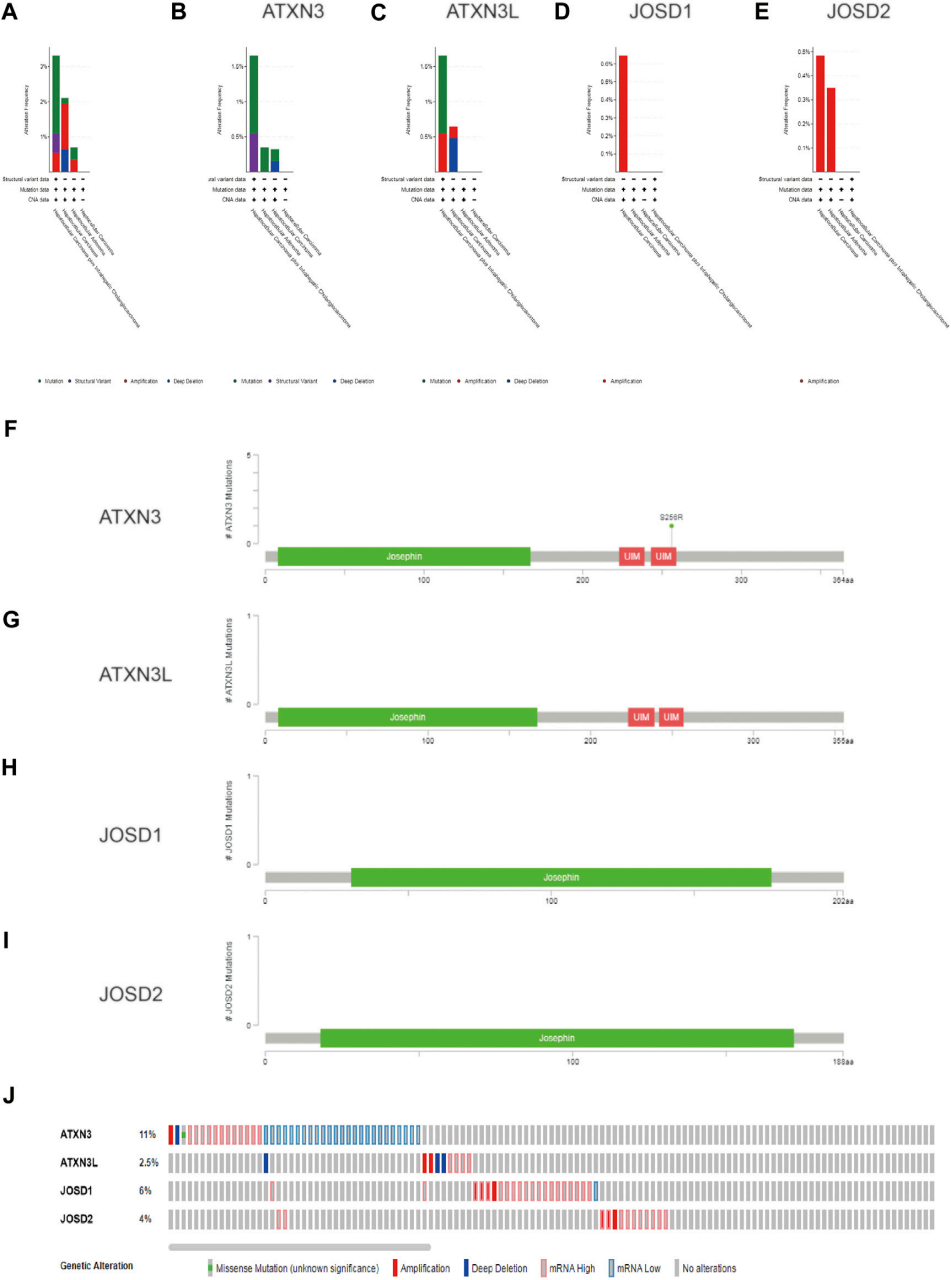
The genetic alteration and mutation of MJDs family members in HCC (cBioPortal) **(A–E)** The proportions of alternations and mutations of MJDs family members were indicated in various subgroups of HCC **(F–I)**. Schematic representation of gene mutation sites of MJDs on the coding strand **(J)**The genetic alternations and mutations of ATXN3, ATXN3L, JOSD1, and JOSD2 were showed in HCC patients **(E)**.

To explore the reasons for the upregulation of mRNA expression levels, we analyzed the structural variation of the MJDs family gene in HCC by the cBioPortal. MJDs family members have different trends in different HCC subclasses. The sub-types of with the highest proprotion of all alterations were Hepatocellular carcinoma plus Intrahepatic Cholangiocarcinoma, Hepatocellular Adenoma, and Hepatocellular carcinoma for ATXN3, ATXN3L, JOSD1, and JOSD2, respectively (Figures 5A–E). Figures 5F–I displayed the specific mutation sites in MJDs DNA sequences, in which the green dots represent missense mutations. Interestingly, Genetic alterations include missense mutation, amplification, deep deletion, and mRNA high or low (Figure 5J), among which 11% ATXN3, 2.5% ATXN3L, 6% JOSD1, and 4% JOSD2 showed structural variation. These results suggested that MJDs family members had considerable genetic stability as potential HCC patients’ diagnostic biomarkers.

**FIGURE 6:**
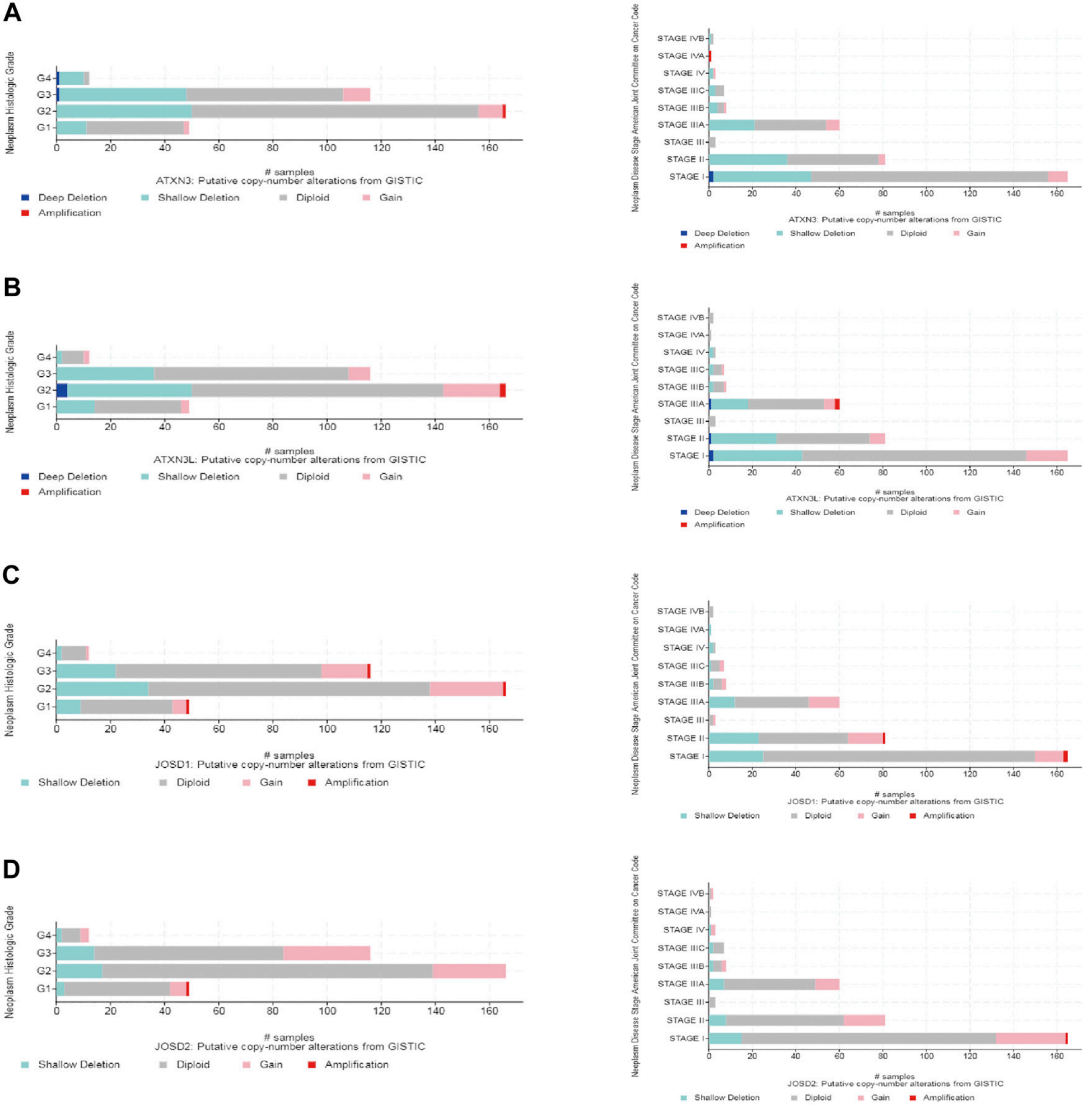
MJDs family members genomic alterations in HCC (cBioPortal) **(A–D)** CNV frequencies of MJDs family members were distributed in different of stages and grades of subgroups.

Moreover, the frequency distribution of MJDs family members’ CNV patients in different stage and grade groups was shown. This suggested a high incidence and early event of the MJDs family members’ CNV alteration in HCC (Figures 6A–D).

### Survival analysis of MJDs family members in HCC

**FIGURE 7:**
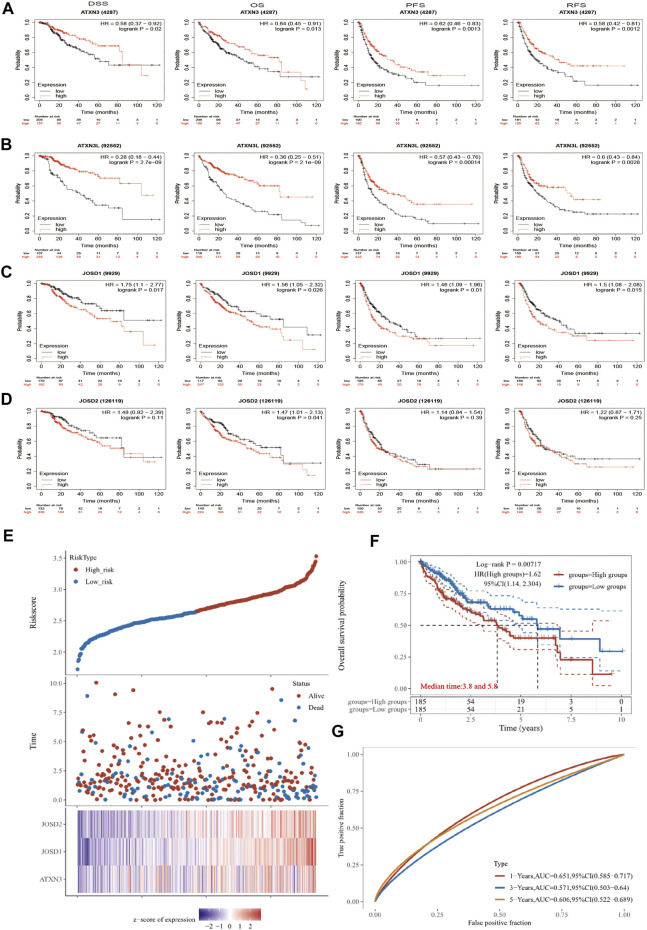
**(A‐G)** The prognosis of MJDs expression level in HCC patients. The HCC specimens were divided into two groups according to the median expression levels of MJDs family members. The disease-specific survival (DSS, n = 362), overall survival (OS, n = 364), progression-free survival (PFS, n = 370), and relapse-free survival (RFS, n = 316) were compared between patients across high and low expression of MJDs family members. TCGA database screening results for 371 HCC patient samples, including risk score, survival time, and survival status. The middle shows the survival matching to the Risk score of various samples, while the top shows the Risk score scatter plot from low to high, with different colors denoting distinct risk categories. Time and survival status scatter plot distribution; the expression heat map of the signature’s genes is shown in the bottom figure **(E)** The KM survival curve distribution of the risk model in the data set, in which different groups are tested by log rank, HR (High risk) represents the risk coefficient between the high-risk group and the low-risk group samples, and HR > 1 indicates that the model is a risk model. 95% CI represents the HR confidence interval **(F)** The risk model’s ROC curve and AUC at various points; the greater the AUC value, the better the model’s capacity for forecasting **(G)**.

To evaluate the prognostic effects of MJDs and MJDs family members’ DNA methylation, we used Kaplan–Meier and MethSurv to explore the correlation between MJDs family members’ mRNA expressions and prognoses in HCC patients. The main parameters of survival analysis include DSS, OS, PFS, and RFS. The data from Kaplan–Meier Plotter was displayed in Figure 7, suggesting that highly expressed ATXN3 and ATXN3L levels were a remarkable correlation with better prognosis in HCC patients. Interestingly, we found that high-expression ATXN3 and ATXN3L had better DSS, OS, PFS, and RFS in HCC patients. On the contrary, high expression levels of JOSD1 and JOSD2 implicated a worse prognosis in HCC patients. Moreover, we found that highly expressed JOSD1 showed a significant association with worse DSS, OS, PFS, and RFS in overall HCC patients. Figure 7 and Table 1 revealed the more detailed prognostic information about MJDs members in HCC.

**TABLE 1:** Prognostic analysis of the MJDs in Kaplan–Meier plotter.

Genes	Expression	DSS		OS		PFS		RFS	
		HR (95% CI)	*p*-value	HR (95% CI)	*p*-value	HR (95% CI)	*p*-value	HR (95% CI)	*p*-value
ATXN3	Upregulated	0.58 (0.37–0.92)	0.02	0.64 (0.45–0.91)	0.013	0.62 (0.48–0.83)	0.0013	0.58 (0.42–0.81)	0.0012
ATXN3L	Upregulated	0.28 (0.18–0.44)	2.7e-09	0.36 (0.25–0.51)	2.1e-09	0.57 (0.43–0.76)	0.00014	0.60 (0.43–0.84)	0.0028
JOSD1	Upregulated	1.75 (1.1–2.77)	0.017	1.56 (1.05–2.32)	0.026	1.46 (1.09–1.96)	0.01	1.50 (1.08–2.08)	0.015
JOSD2	Upregulated	1.48 (0.92–2.39)	0.11	1.47 (1.01–2.13)	0.041	1.14 (0.84–1.54)	0.39	1.22 (0.87–1.71)	0.25

Correlation Between MJDs, Family Members’ Gene Copy Number and Immune Cell Infiltration.

We used dimensionality reduction based on Lasso regression to analyze the prognosis of MJDs members gene on patients with HCC and created a predictive model to assess OS. In Figure 7E, we found that when the three genes of ATXN3, JOSD1, and JOSD2 were co-expressed, the number of patients died and the risk score increased. The Kaplan–Meier Plotter showed that high risk score group of ATXN3, JOSD1, and JOSD2 overall high expression had worse OS, compared with low risk score group (Figure 7F). According to the ROC curve of the risk model established at different times, the AUC of 1-Years, 3-Years, 5-Years was 0.651, 0.571, 0.606, which indicated that the model has strong predictive ability (Figure 7G). This prognostic model indicated that worse survival was associated with overall high expression of MJDs members.

**FIGURE 8:**
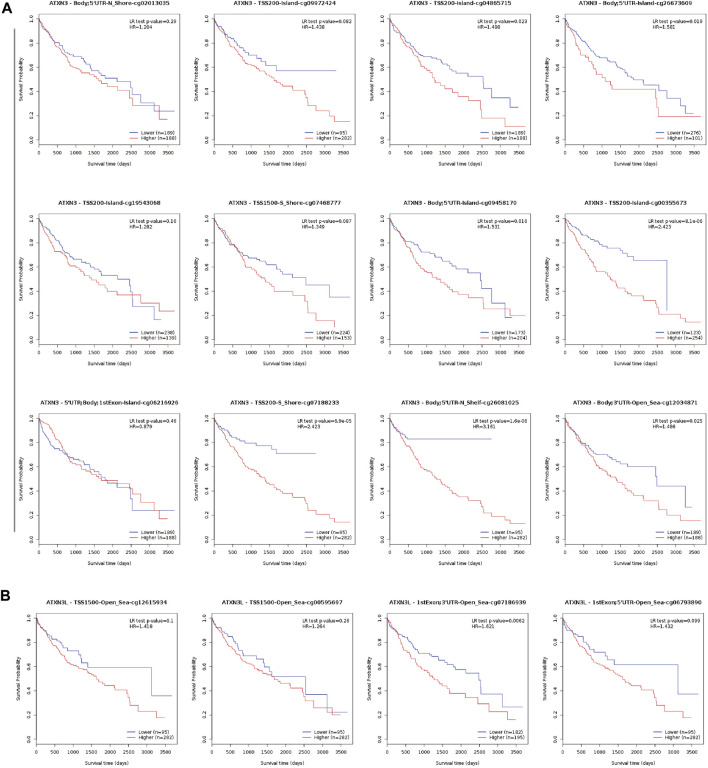
Kaplan-Meier survival curves comparing the high or low expression of MJDs methylation sites in HCC (MethSurv). Kaplan-Meier survival analysis of **(A)** ATXN3 and **(B)** ATXN3L methylation sites were shown.

Subsequently, the MethSurv database was used to further reveal the relationship between methylation sites of MJDs and prognosis in HCC patients (Figure 8, Supplementary Figure S3). We found that the methylation level of cg26081025 in ATXN3 (HR = 3.161, *p*-value = 1.6e-06), cg07186939 in ATXN3L (HR = 1.621, *p*-value = 0.0062), cg25697769 in JOSD1(HR = 1.828, *p*-value = 0.004), and cg18708810 in JOSD2(HR = 1.735, *p*-value = 0.012) were a risk factor on survival. However, the methylation level of cg06711259 (HR = 0.455, *p-*value = 3.1e-05), cg19658332 (HR = 0.612, *p*-value = 0.014) cg27610821 (HR = 0.665, *p*-value = 0.023), and cg01138530 (HR = 0.67, *p*-value = 0.026) in JOSD1 were a protective factor on survival. Finally, the results suggested that eight CpG sites in ATXN3, the one CpG site in ATXN3L, 10 CpG sites in JOSD1 and the one CpG site in JOSD2 were related to the prognosis in HCC patients (Figure 8, Supplementary Figure S3, and Supplementary Table S2).

MJDs family members may regulate the development, distribution, and maturation of immune cells, but the correlation between MJDs family members’ expression levels and immune-cell infiltration in HCC remains poorly unclear.

**FIGURE 9:**
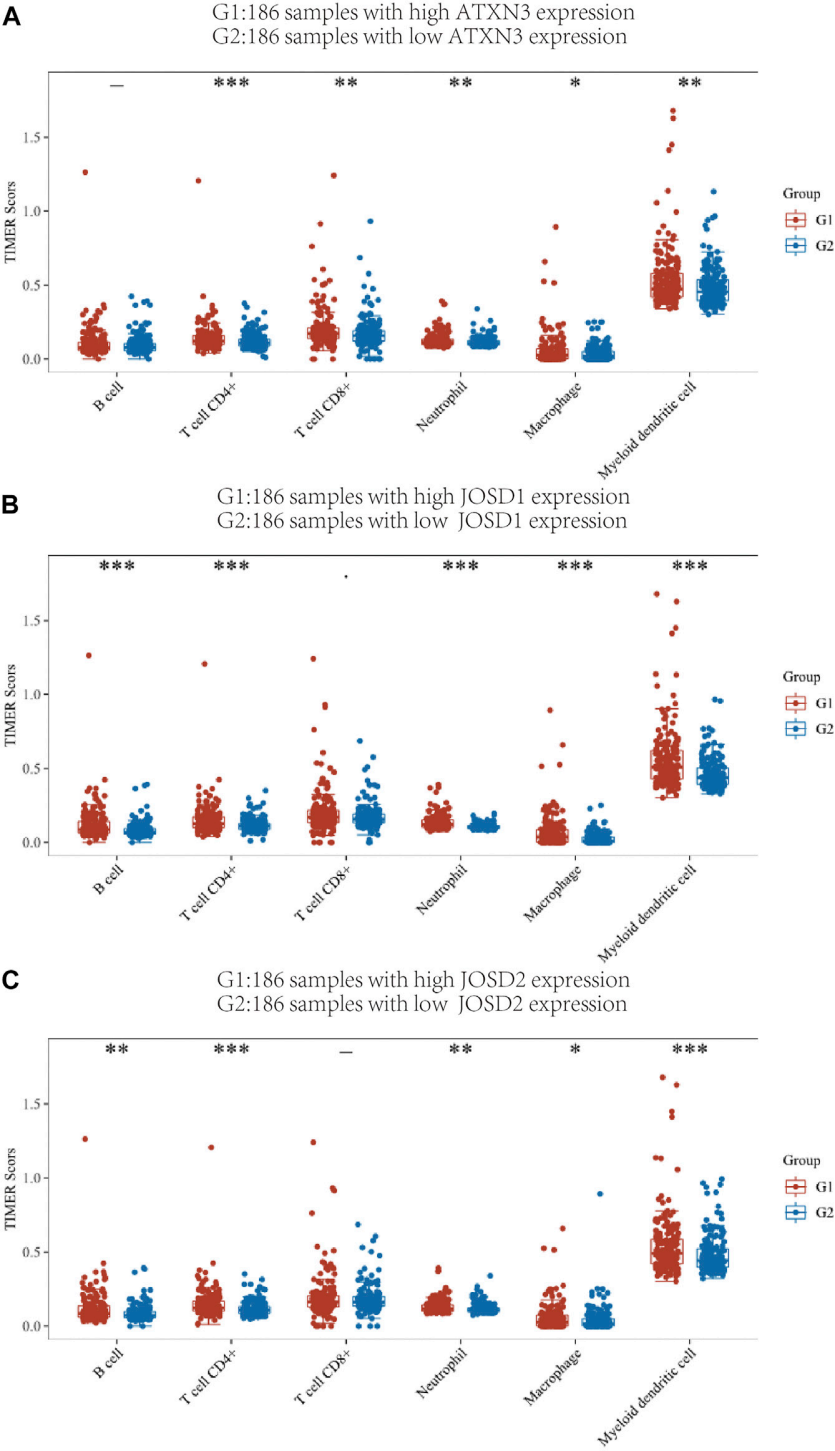
TIMER immune score of high expression group and low expression group of MJDs gene **(A‐C)** Different colors represent different immune cell types, the abscissa represents the group, and the ordinate represents the percentage of immune cells in a single group. This graph compares the percentage abundance of tumor-infiltrating immune cells in the high expression group (G1) and low expression group (G2) of MJDs genes. *p < 0.05, **p < 0.01, and ***p < 0.001.

We used the TIMER score to assess this correlation. According to the expression levels of the ATXN3, JOSD1, and JOSD2 genes, the HCC patient samples in the TCGA database were split into 186 high-expression groups and 185 low-expression groups. In Figure 9A, the high-expression ATXN3 group had higher expression of CD4+T cells, CD8+T cells, macrophages, myeloid dendritic cells, B cells, and neutrophils, compared with the low-expression ATXN3 group. Then, JOSD1 expression was positively correlated with B cells, CD8+T cells, CD4+T cells, macrophages, neutrophils, and dendritic cells (Figure 9B). In addition, the change in JOSD2 expression was positively correlated with B cells, CD8+T cells, CD4+T cells, macrophages, neutrophils, and dendritic cells (Figure 9C). Therefore, these results implicated that MJDs family members expression may predict the infiltration of various kinds of immune cells in HCC.

In addition, correlations between the MJDs family members’ somatic copy number alterations (SCNA) and tumor immune infiltration levels in HCC patients were explored. The study showed that the SCNA of ATXN3 and JOSD1 had no significant correlations with the infiltration levels of CD4^+^ T cells, CD8^+^ T cells, B cells, neutrophils, macrophages, and DCs; the SCNA of ATXN3L had significant correlations with CD4^+^ T cells, CD8^+^ T cells, B cells, neutrophils, macrophages, and DCs; moreover, the SCNA of JOSD2 had significant correlations with CD4^+^ T cells (Supplementary Figure S4). These findings revealed that changes in the SCNA of the ATXN3L and JOSD2 may reflect tumor infiltration levels in HCC.

### Relationship between MJDs members and immune molecules

**FIGURE 10:**
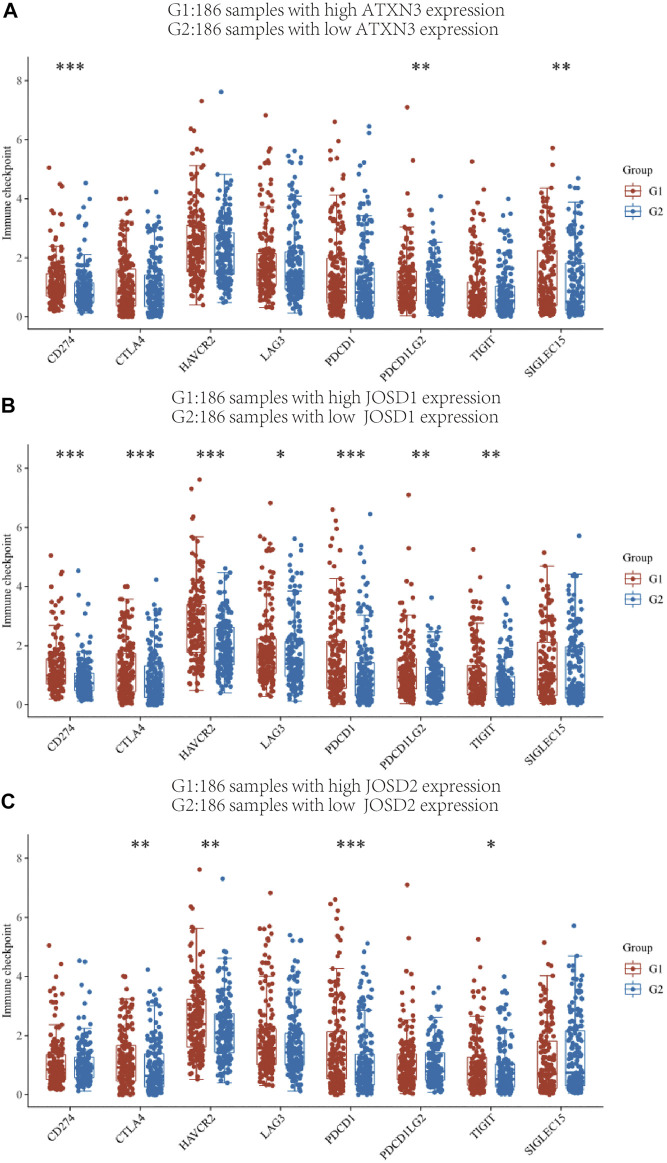
The relationship between MJDs gene expression and immune checkpoints **(A‐C)** The HCC patients in the TCGA database were classified into high expression group (G1) and low expression group (G2) based on the various levels of ATXN3, JOSD1 and JOSD2 gene expression, and the association between the two groups and the expression of immune checkpoints was presented, respectively. *p < 0.05, **p < 0.01, and ***p < 0.001.

The link between MJDs members and immune checkpoint molecules was then thoroughly investigated. The HCC patient samples in the TCGA database were divided into high and low expression groups of ATXN3, JOSD1, JOSD2, and the distribution of immune checkpoints in different gene expression samples was studied (Figure 10). The gene expression of ATXN3 was positively correlated with the expression of CD274 (*p*<0.001), PDCD1LG2 (*p* < 0.01), SIGLEC15 (*p*<0.01); the gene expression of JOSD1 had positive correlations with CD274 (*p*<0.001), CTLA4 (*p* < 0.001), HAVCR2(*p* < 0.001), LAG3 (*p* < 0.05), PDCD1 (*p* < 0.001), PDCD1LG2 (*p* < 0.01), TIGIT (*p* < 0.01); and the gene expression of JOSD2 had positive correlations with CTLA4 (*p* < 0.01), HAVCR2(*p* < 0.01), PDCD1 (*p* < 0.001),TIGIT (*p* < 0.05). These findings showed a positive correlation between the expression of immunological checkpoints and the elevation of MJDs member genes.

**FIGURE 11:**
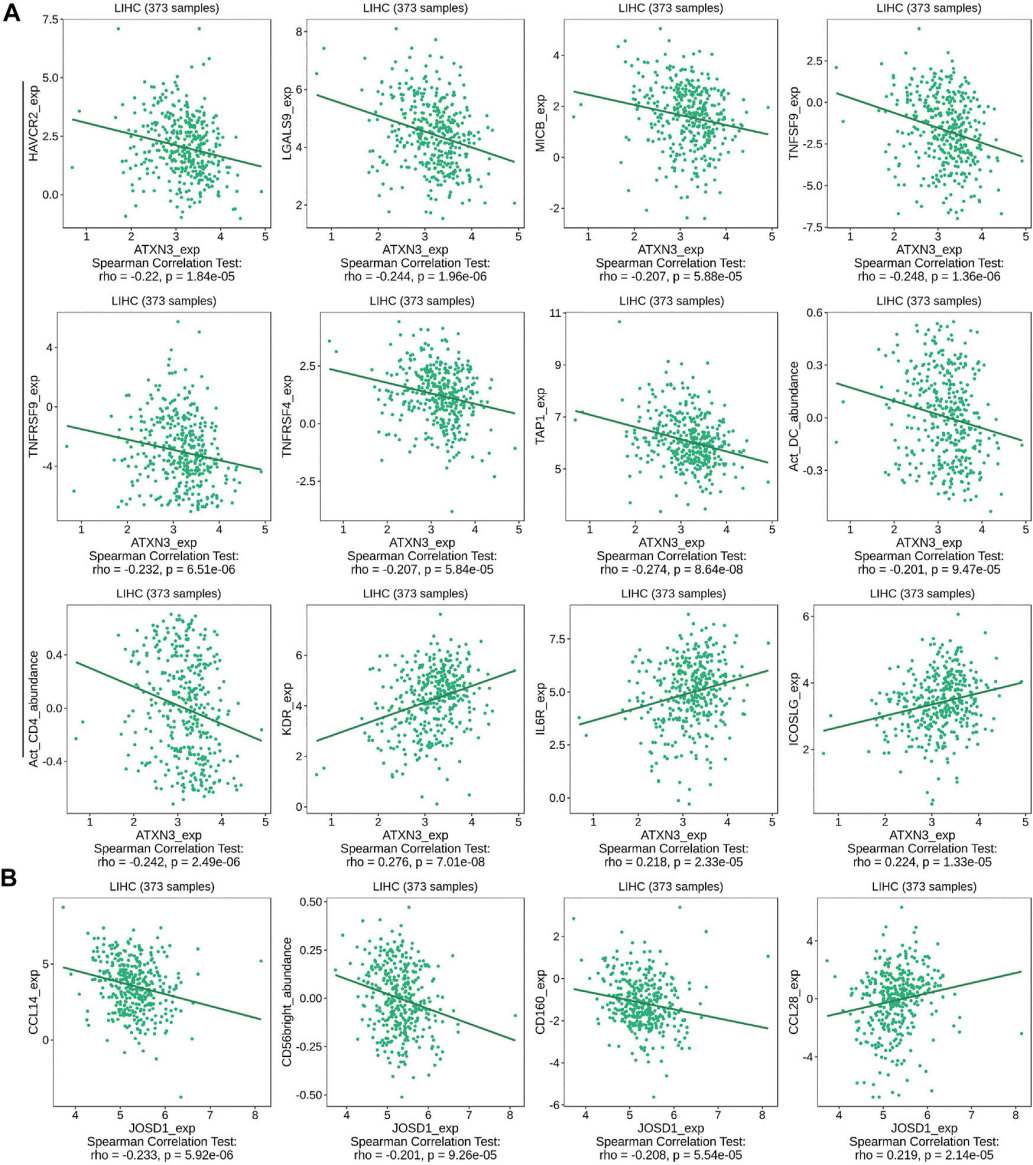
Correlation between MJDs level and lymphocytes, immunomodulators, and chemokines expression in HCC from TISIDB database. The correlation between alterations in expression levels of **(A)** ATXN3, and **(B)** JOSD1 with the expression of immune cells marker in HCC were performed.

We also explored relationships between MJDs expression and various immune signatures with the TISIDB database. Associations between MJDs expression and different immune markers were obtained from the TISIDB database. Figure 11 and Supplementary Figure S5 showed the correlations between MJDs and TILs which included MHC-TAP1, Act_CD4_abundance, Act_DC_abundance, and CD56bright. Immunomodulators can be divided into three groups: immunoinhibitors, immunostimulators, and MHC molecules. In Figure 11A and Table 2, HAVCR2, LGALS9, MICB, TNFRSF4, TNFRSF9, TNFSF9, MHC_TAP1, Act_CD4, and Act_DC were negatively associated with the ATXN3 expression, while KDR, IL6R, and ICOSLG positively correlated with the ATXN3 expression. In Figure 11B and Supplementary Table S3, JOSD1 negatively correlated with CD160, CD56bright, and CCL14, while CCL28 was positively related to this gene. In the Supplementary Figure S5 and Supplementary Table S4, JOSD2 significantly positively correlated with LGALS9, PVRL2, TNFRSF4, TNFRSF14, TNFRSF18, TNFRSF25, MHC_HLA-A, and MHC_HLA-DMA, while CD274, CD28, CCR4, IL6R, KDR, and TGFBR1 negatively correlated with JOSD2 expression. Thus, these results demonstrated that ATXN3, JOSD1, and JOSD2 were related to various immune molecules in HCC, which implicated a significant role in immune escape.

**TABLE 2:** Correlation of ATXN3 expression with immunomodulators based on TISIDB database.

Immunomodulators	ATXN3 expression TISIDB rho, n = 373	*P*
HAVCR2	-0.22	1.84e-05
KDR	0.276	7.01e-08
LGALS9	-0.244	1.96e-06
ICOSLG	0.224	1.33e-05
IL6R	0.218	2.33e-05
MICB	-0.207	5.88e-05
TNFRSF4	-0.207	5.84e-05
TNFRSF9	-0.232	6.51e-06
TNFSF9	-0.248	1.36e-06
TAP1	-0.274	8.64e-08
Act_CD4	-0.242	2.49e-06
Act_DC	-0.201	9.47e-05

### Functions and pathways for MJDs family members in HCC

**FIGURE 12:**
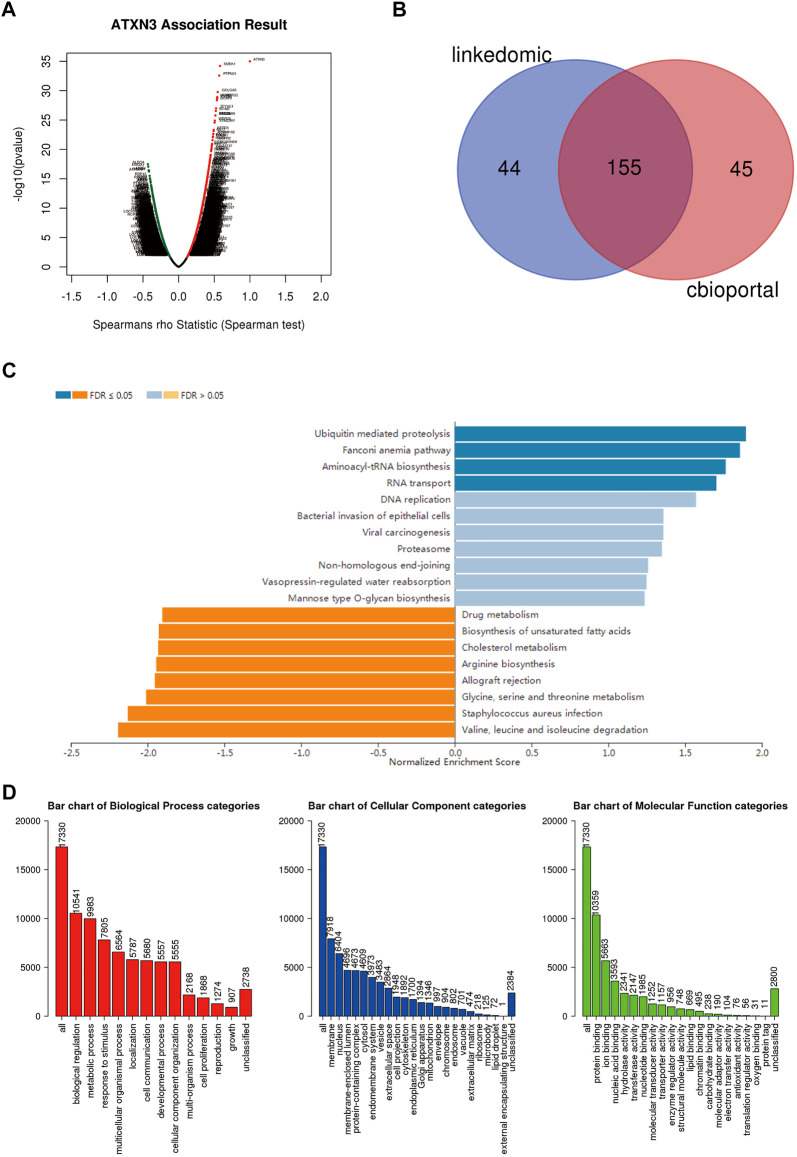
Different genes and pathways connected to MJDs family members expression in HCC **(A)** Volcano plot suggested that the differential expression of genes correlated with ATXN3 in HCC **(B)** The top 200 co-expressed genes were extracted from the LinkedOmics and cBioPortal, and then the above genes were intersected **(C,D)** The prediction of GO analysis and KEGG pathway of MJDs family members were showed.

We used the LinkedOmics database to analyze the differentially expressed genes associated with ATXN3, JOSD1, and JOSD2 in HCC (Figure 12A, Supplementary Figures S6A,7A). LinkedOmics and the cBioPortal database were screened for the top 200 co-expressed genes to obtain crossover genes, respectively (Figure 12B, Supplementary Figure S6B,7B). The above intersecting genes were obtained and analyzed by GO and KEGG to predict functions and pathways of MJDs family members. The KEGG pathways showed that for ATXN3, ubiquitin mediated proteolysis, fanconi anemia pathway, aminoacyl-tRNA biosynthesis and RNA transport were significantly up-regulated, whereas valine, leucine and isoleucine degradation, *staphylococcus aureus* infection, glycine serine and threonine metabolism were significantly down-regulated (Figure 12C). For JOSD1, allograft rejection, Th17 cell differentiation, and tryptophan metabolism were significantly up-regulated, whereas DNA replication and fanconi anemia pathway were significantly down-regulated (Supplementary Figure S6C). For JOSD2, allograft rejection, Th17 cell differentiation, and ascorbate and aldarate metabolism were significantly up-regulated, whereas ribosome, oxidative phosphorylation, and protein export were significantly down-regulated (Supplementary Figure S7C). Interestingly, GO analysis showed that the co-expressed genes of MJDs family members were the same in Biological process (BP), Cellular component (CC) and Molecular Function (MF). Specifically, BP terms indicated regulation, metabolic process, and response to a stimulus. CC terms implicated the membrane, nucleus, and membrane-enclosed lumen. MF terms suggested protein binding, ion binding, and nucleic acid binding (Figure 12D, Supplementary Figure S6D,7D).

Furthermore, we analyzed the co-expression of ATXN3, JOSD1 and JOSD2 in hepatocellular carcinoma using the GSCALite database, and analyzed the signaling pathways involved by MJDs family member respectively (Supplementary Figure S8). We found that ATXN3 inhibited apoptosis, while JOSD1 and JOSD2 promoted apoptosis, JOSD2 inhibited DNA damage response, JOSD1 and JOSD2 promoted EMT, and JOSD2 suppressed RTK and androgen pathways.

Moreover, we used the cBioPortal database to explore the co-expression (Table 3) and mutual exclusion (Table 4) between individual members of the MJDs family. Our predicted results showed that there was no co-expression and no mutual exclusion among MJDs family members, but further experiments such as immunoprecipitation or immunofluorescence were needed to verify.

**TABLE 3:** Co-expression of MJDs family members

Gene	Correlated gene	Cytoband	Spearman’s correlation	*p*-Value	*q*-Value
ATXN3L	JOSD2	19q13.33	0.0872	0.0984	0.446
	ATXN3	14q32.12	0.0319	0.546	0.848
	JOSD1	22q13.1	0.0953	0.071	0.388
ATXN3	JOSD1	22q13.1	-0.0154	0.771	0.851
	JOSD2	19q13.33	-0.108	**0.04**	0.0905
JOSD1	JOSD2	19q13.33	-0.0351	0.507	0.623

**TABLE 4:** Mutual exclusivity of MJDs family members.

Gene A	Gene B	Neither	A not B	B not A	Both	Log2 Odds ratio	*p*-Value	*q*-Value	Tendency
ATXN3	JOSD1	299	39	21	1	-1.454	0.272	0.655	Mutual exclusivity
JOSD1	JOSD2	325	22	13	0	<-3	0.434	0.655	Mutual exclusivity
ATXN3	JOSD2	309	38	11	2	0.564	0.434	0.655	Co-occurrence
ATXN3L	JOSD1	330	8	21	1	0.974	0.437	0.655	Co-occurrence
ATXN3	ATXN3L	312	39	8	1	0	0.658	0.715	Mutual exclusivity
ATXN3L	JOSD2	338	9	13	0	<-3	0.715	0.715	Mutual exclusivity

### Validation of the expression of MJDs family members in HCC tissue and HCC cell lines

**FIGURE 13:**
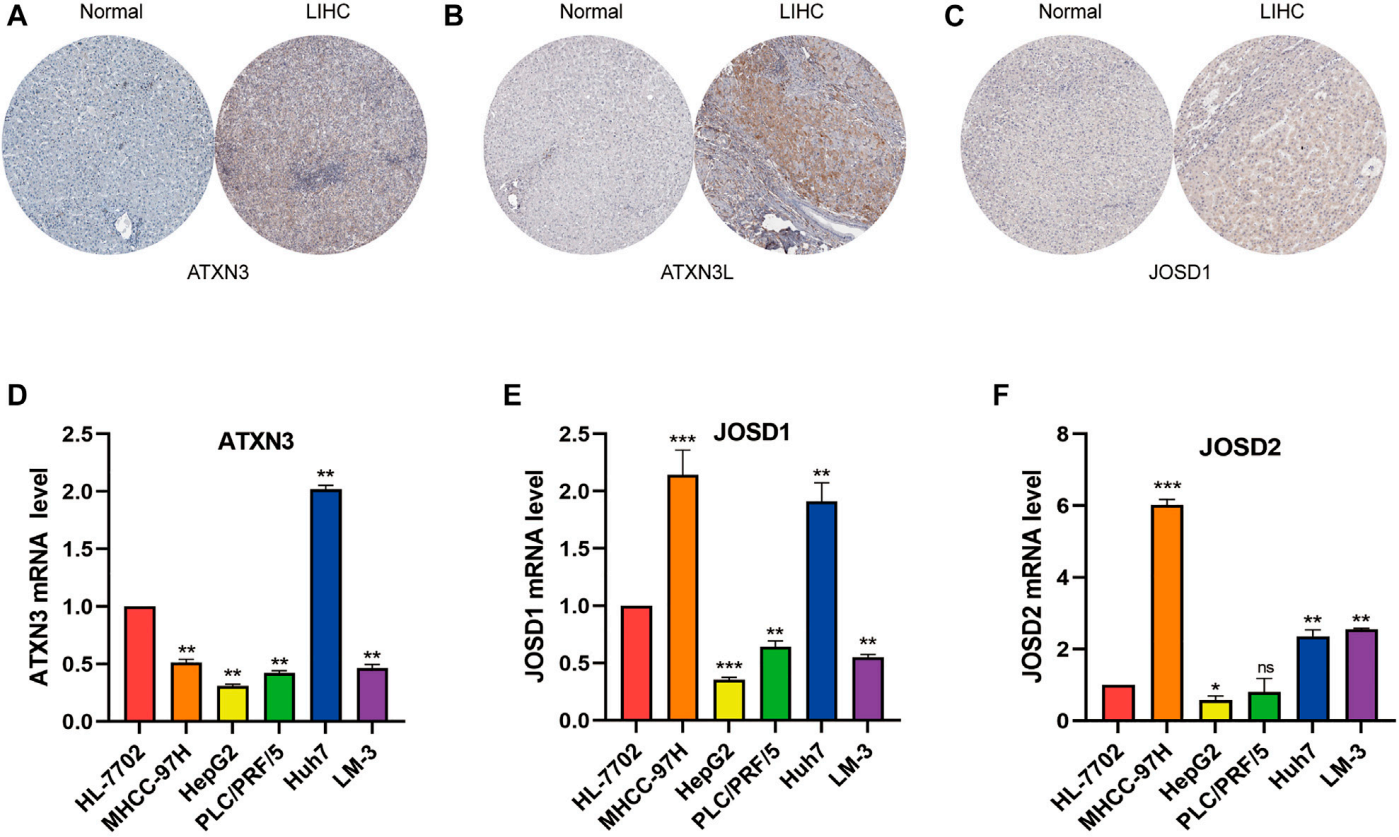
The expression levels of MJDs family members in HCC tissue and cell lines. The protein expression of **(A)** ATXN3, **(B)** ATXN3L, and **(C)** JOSD1 in HCC and normal liver tissues were obtained by immunohistochemical (IHC) data in the HPA database. The IHC image of JOSD2 was absent. The expression levels of **(D)** ATXN3, **(E)** JOSD1, and **(F)** JOSD2 in human immortalized HL-7702 and different HCC cell lines were conformed using RTqPCR.Ct values of ATXN3L were absent in HL-7702, MHCC-97 and HepG2 cell lines. *p < 0.05, **p < 0.01, and ***p < 0.001.

Next, we explored the MJDs family members’ expression levels between HCC samples and normal liver samples from the HPA database. The IHC staining of MJDs family members showed that the mRNA expression levels of ATXN3, ATXN3L, and JOSD1 were enhanced compared with the liver tissues (Figures 13A–C), which consistent with above results. The IHC staining of JOSD2 was pending HCC tumor tissue analysis in the HPA.

To verify above results with bioinformatics analysis, we used qRT-PCR to confirm the expression levels of MJDs in five HCC cell lines which included MHCC-97H, HepG2, PLC/PRF/5, Huh7, and HCCLM-3, respectively, compared with the HL-7702. The results suggested that, except ATXN3L, the expression levels of MJDs were elevated in multiple HCC cell lines (Figure 13 D-F), in consistent with the above results, and further experiments were needed to support it.

## Discussion

Accumulating evidence indicated that the abnormal expression or mutation of deubiquitinases (DUBs), with the function of promoting the occurrence and development of malignant tumors, was emerging as therapeutic targets of cancer (Lv et al., 2020; Park and Baek, 2022). MJDs family, consisting of ATXN3, ATXN3L, JOSD1, and JOSD2, was the smallest family of the DUBs (Harrigan et al., 2018). MJDs family had a common Josephin domain, containing around 180 amino acids. According to recent studies, MJDs family members were aberrant expressions in some cancer and were related to biological functions and prognoses in cancers (Zeng et al., 2020). However, the aberrant mRNA expressions of MJDs family members and their biological functions and mechanisms in HCC were poorly understood. Thus, we further explored the role of MJDs family members in HCC, particularly, including immune microenvironment and promoter methylation levels.

Recent studies reported that oral squamous cell carcinoma (OSCC) (Song et al., 2021), testicular cancer (TC) (Shi et al., 2018), colon cancer (Li et al., 2019), breast cancer (BC) (Zou et al., 2019), anaplastic thyroid carcinoma (ATC) (Zhuang et al., 2021), neuroblastoma (NB) (Gong et al., 2021), non-small cell lung cancer (NSCLC) (Sacco et al., 2014), and breast cancer stem cells (Zhu et al., 2019)were closely related to ATXN3. For example, for oral squamous cell carcinoma, it had been studied that down-regulated miR-619-5p promoted the proliferation, migration, and invasion abilities by enhancing ATXN3 expression in OSCC cisplatin-resistant cells. The miR-619-5p/ATXN3 axis provided the potential molecular mechanism of OSCC, especially for promoting drug resistance of cisplatin of OSCC. And ATXN3 may be a putative therapeutic target for OSCC patients (Song et al., 2021). In addition, ATXN3 knockdown can inhibit cell growth. Meanwhile, down-regulated ATXN3 can promote PTEN expression and inactivate the AKT/mTOR pathway (Shi et al., 2018). Previous research reported that ATXN3L was expressed at higher levels in breast cancer. ATXN3L directly interacted with KLF5, thus, reducing KLF5’s ubiquitination and degradation (Ge et al., 2015). JOSD1 was found to be up-regulated in the head and neck squamous cell carcinoma (HNSCC) (Jing et al., 2021), gynaecological cancer (GC) cells (Wu et al., 2020), and acute myeloid leukemia (AML) cells (Yang et al., 2022). Over-expressed JOSD2 was positively correlated with the worse prognosis in hepatocellular carcinoma (Huang et al., 2022), cholangiocarcinoma (CCA) (Qian et al., 2021), and NSCLC (Krassikova et al., 2021).

Our results showed that ATXN3, JOSD1, and JOSD2 were markedly up-regulated in HCC samples, while ATXN3L was expressed only in testicular and testicular tumors. We found that the higher mRNA expression levels of JOSD1 and JOSD2 in HCC patients had a significantly worse prognosis. Interestingly, we analyzed that the prognostic value of MJDs family members’ expression levels in HCC patients revealed that higher mRNA expression of AXTN3 and ATXN3L have better OS, DSS, PFS, and RFS, which was not consistent with previous studies. The prognostic model indicated that worse survival was associated with overall high expression of MJDs members. Furthermore, expression levels of ATXN3, JOSD1, and JOSD2 were significantly related to different clinicopathological parameters in HCC. Thus, the above results strongly revealed that ATXN3, JOSD1, and JOSD2 deserved further investigation of potential targets in HCC.

Many studies indicated that methylation levels were closely correlated with tumor development, cancer progression, and chemotherapy and immunotherapy sensitivity of HCC (Al-Abdulla et al., 2019; Dong et al., 2021; Zhong et al., 2021; Liu et al., 2022) Thus, to explore the mechanism for the aberrant expression of MJDs family members in HCC, we further analyzed the correlations between these genes expression levels and promoter methylation levels with the UALCAN database. In our study, the promoter methylation levels of ATXN3 and JOSD2 were lower expressed in these genes’ high mRNA expression group in TCGA HCC samples, while JOSD1 was highly expressed in the JOSD1 high expression group. Moreover, we used the cBioPortal database to analyze the correlations between promoter methylation degrees of ATXN3, JOSD1, and JOSD2 and the transcriptional levels. We found that the Spearman coefficients of ATXN3, JOSD1, and JOSD2 were -0.31,-0.35, and -0.29, respectively, suggesting significantly negative correlations, which was consistent with the above results. In addition, it was worth noting that the promoter methylation levels of ATXN3L were significantly lower in HCC tissues than in normal tissues, and the methylation β value of ATXN3L was greater than 0.6, presenting a full methylation status. Furthermore, the previous research showed that the methylation mediated inactivation of genes was common in HCC compared with normal samples and connected to clinical features and prognose (He et al., 2020; Xu et al., 2021). Consistently, our results demonstrated that promoter methylation levels of MJDs family members were up-regulated in clinical features of HCC. Meanwhile, we further analyzed relationships of 39 loci within MJDs family members’ prognosis and confirmed these genes’ DNA methylation of twenty loci located on promoter CpG islands, which connected to worst survival. The above results indicated that abnormal methylation might be one of the crucial reasons for the aberrant expression of MJDs gene family. However, other epigenetic modifications, involving gene mutations, CNV, and SNV might also play important roles in the abnormal expression of these genes.

To further elucidate the potential mechanism of the MJDs family, co-expressed genes with ATXN3, JOSD1, and JOSD2 in HCC samples from the TCGA database were extracted for GO and KEGG enrichment analysis to study the functions of the MJDs family in HCC. As expected, the biological functions of MJDs family members primarily correlated with ubiquitin mediated proteolysis, Th17 cell differentiation, valine, leucine and isoleucine degradation, glycine serine and threonine metabolism, and oxidative phosphorylation. It was reported that MJDs family members involve multiple signaling pathways, including ubiquitin pathway (Zhuang et al., 2021), apoptosis (Li et al., 2019), Hedgehog signaling pathway (Xu et al., 2021), JAK2 (Yang et al., 2022), and Akt/PI3K signaling (Sacco et al., 2014). In addition, a previous study reported that the serine-biosynthesis pathway and tyrosine-relative kinase inhibitors played important regulatory roles in tumor progression and immunotherapy in liver cancer patients (Sangro et al., 2021). These findings suggested that ATXN3, JOSD1, and JOSD2 could participate in the progression of HCC through primary regulating genes post-translational modification pathways, and the immune environment.

It was critical to investigate the relationships between MJDs family members’ expression levels and the multiple immune-cell infiltration in HCC. Based on the TIMER database, obviously, correlations were shown between MJDs family members’ expression and the infiltrating levels of B cells, CD4^+^ T cells, CD8^+^ T cells, neutrophils, dendritic cells (DCs), and macrophages. Relevant evidence had shown that the presence of tumor-infiltrating lymphocytes (TILs) might relate to the prognosis of HCC patients (Li et al., 2020; Nie et al., 2021). Immune cell based immunotherapy (Liu et al., 2021), including T Cells (Woller et al., 2021), neutrophils (Geh et al., 2022), and dendritic cells (Matsui et al., 2021), played crucial roles in the immunological therapy of HCC. In general, previous studies and our research revealed that MJDs family members might be involved in the regulation of the infiltration cell recruitment of immune cells in the immune microenvironment, which could be targeted for immunotherapy in HCC patients.

The combination of immune therapy and the classic target therapies may be the emerging trend of clinical comprehensive treatment. For our study, we revealed that the transcriptional expression of MJDs family members was correlated with lymphocytes, immunomodulators (immunomoinhibitor and immunomostimulator), and chemokines in HCC from the TISIDB database. In this research, HAVCR2, LGALS9, MICB, Act_CD4, and Act_DC were negatively associated with the ATXN3 expression, while KDR, IL6R, and ICOSLG positively correlated with the ATXN3 expression. A recent study identified the binding of HAVCR2 to LGALS9 contributes to Th1 cell death via apoptosis (Zhu et al., 2005). Meanwhile, HAVCR2 was an inducible NK cell receptor that promotes IFN-γ production in response to LGALS9 (Gleason et al., 2012). Tocilizumab, an IL6R antagonist, was approved for the management of CAR T-cell-related Cytokine Release Syndrome (Patel et al., 2021). Resistance to checkpoint blockade is mainly mediated via down-regulated expression of MHC-I in tumor cells. The MHC -I chain-related polypeptide A/B (MICA/B), expressed in many human cancers, serves as ligands to activate the NKG2D receptor on the NK cells and T cells (Raulet et al., 2013). Inhibition of proteolytic shedding of MICA/B leads NK cells mediated immunity against tumors resistant to T cells (Ferrari de Andrade et al., 2020). Similarly, JOSD1 negatively correlated with CD160 and CCL14, while CCL28 was positively related to this gene. In addition, JOSD2 positively correlated with TNFRSF18, HLA-A, and HLA-DMA, while CD274, TGFBR1, CCR4, and CD28 negatively correlated with JOSD2 expression. Emerging studies have found that chemokine ligands (CCL) and chemokine receptors (CCR) are closely connected to the immune therapy of HCC. Notably, CCL14 expression in HCC negatively correlated with PD-1, HAVCR2, and CTLA-4, suggesting its role in regulating tumor immunity (Gu et al., 2020). CCR4 could enhance anti-tumor immunity, mainly by targeting and blocking the infiltration of regulatory T cells (Tregs)into the tumor microenvironment and inhibiting the stability of the TIL-Treg pool (Gao et al., 2022). Lenvatinib showed lower CD274(PD-L1) expression and Tregs infiltration in recurrent HCC compared with primary HCC(Yi et al., 2021). It has been excitingly reported that methylation at certain CpG sites was an indicator of immune infiltration of cancer and might predict patient response to checkpoint inhibitors (Bacolod et al., 2019). Combining previous studies with our results suggested that ATXN3, JOSD1, and JOSD2 might be a scheme to improve the outcome of immunotherapy. Therefore, it was necessary to further explore more detailed mechanism of transcriptional levels/DNA methylation of MJDs family members in regulating the tumor micro-environment.

In summary, our results suggested that the MJDs family members’ aberrant expression in HCC played an important role in clinical features, prognosis, tumor micro-environment, immune-related molecules, mechanisms, and functions. Therefore, our study will give support to elucidate the clinical significance of MJDs family members’ expression levels and DNA methylation in HCC, and further explore the mechanisms of the MJDs family member’s roles in HCC, and especially provide insight into potentially therapeutic targets to improve HCC patient prognosis. In addition, we found that the online tools were based on different algorithms, leading to inconsistent analysis results. Thus, our results need to be further verified in experiments.

## Data Availability

The original contributions presented in the study are included in the article/Supplementary Material, further inquiries can be directed to the corresponding author.
