# Supplementary material for: MJDs family members: Potential prognostic targets and immune-associated biomarkers in hepatocellular carcinoma
Source: Front Genet. 2022 Sep 9;13:965805. doi: 10.3389/fgene.2022.965805 (PMC9500549; doi:10.3389/fgene.2022.965805)
Supplement: Supplementary file 1 [file DataSheet1.ZIP › Data sheet1/supplement figure/supplement figure legend.docx]

**SUPPLEMENT FIGURE 1** **(A-D)** Bulk tissue gene expression levels of MJDs family members in different types of cells.

**SUPPLEMENT FIGURE 2** Visualization between the methylation level and MJDs expression. **(A-D)** Unsupervised clustering analysis of copy number changes in MJDs methylation sites. Clinical information correlated with the samples were displayed in the column header. Red suggested copy number gain, and blue suggested copy number loss.

**SUPPLEMENT FIGURE 3** Kaplan-Meier survival curves comparing the high or low expression of MJDs methylation sites in HCC (MethSurv). Kaplan-Meier survival analysis of **(A)** JOSD1 and **(B)** JOSD2 methylation sites were shown.

**SUPPLEMENT FIGURE 4** Association between MJDs family members expression and immune-cell infiltration in HCC. The association between genes expression alterations of **(A)** ATXN3, **(B)** ATXN3L, **(C)** JOSD1 and **(D)** JOSD2 with the infiltration levels of B cells, CD8+ T cells, CD4+ T cells, macrophages, neutrophils, and dendritic cells in HCC were shown. **p* < 0.05, ***p* < 0.01, and ****p* < 0.001

**SUPPLEMENT FIGURE 5** Correlation between MJDs level and lymphocytes, immunomodulators, and chemokines expression in HCC from TISIDB database. The connection between alters the expression level of JOSD2 with the expression of immune cells marker in HCC were performed.

**SUPPLEMENT FIGURE 6** Different genes and pathways connected to MJDs family members expression in HCC. **(A)** Volcano plot suggested that the differential expression of genes correlated with JOSD1 in HCC. **(B)** The top 200 co-expressed genes were extracted from the LinkedOmics and cBioPortal, and then the above genes were intersected. **(C, D)** The prediction of GO analysis and KEGG pathway of MJDs family members were showed.

**SUPPLEMENT FIGURE 7** Different genes and pathways connected to MJDs family members expression in HCC. **(A)** Volcano plot suggested that the differential expression of genes correlated with JOSD2 in HCC. **(B)** The top 200 co-expressed genes were extracted from the LinkedOmics and cBioPortal, and then the above genes were intersected. **(C, D)** The prediction of GO analysis and KEGG pathway of MJDs family members were showed.

**SUPPLEMENT FIGURE 8** Signaling pathways implicated in MJDs family members in HCC (GSCALite).
