# Supplementary material for: MJDs family members: Potential prognostic targets and immune-associated biomarkers in hepatocellular carcinoma
Source: Front Genet. 2022 Sep 9;13:965805. doi: 10.3389/fgene.2022.965805 (PMC9500549; doi:10.3389/fgene.2022.965805)
Supplement: Supplementary file 1 [file DataSheet1.ZIP › Data sheet1/supplement figure/Supplement table 1.docx]

**Supplement TABLE 1 MJDS family primer bank index**

| Gene | Forward Primer | Reverse Primer |
| --- | --- | --- |
| ATXN3 | 5’-TGTGCTCAACATTGCCTGAAT-3’ | 5’-GCTGCTGTAAAAACGTGCGATA-3’ |
| ATXN3L | 5’-AGCAATGCCTTGAAGTTCTGG-3’ | 5’-TTGTTGTTGTAATCGAGCCAAGA-3’ |
| JOSD1 | 5’-GGGATACGCTGCAAGAGATTT-3’ | 5’-CCATGACGTTAGTGAGGGCA-3’ |
| JOSD2 | 5’-CGGCAACTATGATGTCAATGTGA-3’ | 5’-GGCAGGTTCAGGATCAGCC-3’ |
| GAPDH | 5’-GGAGCGAGATCCCTCCAAAAT-3′ | 5’-GGCTGTTGTCATACTTCTCATGG-3′ |

**Supplement TABLE 2 Univariable Cox proportional hazards regression survival analyses of different MJDs DNA methylation loci in TCGA LIHC HumanMethylation450K platform**

| Gene | VarNames | UCSC_RefGene_Group | HR | *p-*Value |
| --- | --- | --- | --- | --- |
| ATXN3 |  |  |  |  |
|  | cg09458170 | Body 5'UTR | 1.531 | 0.016 |
|  | cg07468777 | TSS1500 S_Shore | 1.349 | 0.087 |
|  | cg19543068 | TSS200 island | 1.282 | 0.16 |
|  | cg26673609 | Body 5'UTR | 1.581 | 0.019 |
|  | cg04865715 | TSS200 island | 1.498 | 0.023 |
|  | cg09972424 | TSS200 island | 1.438 | 0.082 |
|  | cg02013035 | Body 5'UTR | 1.204 | 0.29 |
|  | cg12034871 | Body 3'UTR | 1.486 | 0.025 |
|  | cg26081025 | Body 5'UTR N_Shelf | 3.161 | 1.60E-06 |
|  | cg07188233 | TSS200 S_Shore | 2.423 | 6.90E-05 |
|  | cg06216926 | 5'UTR;Body;1stExon Island | 0.879 | 0.46 |
|  | cg00355673 | TSS200 island | 2.423 | 8.10E-06 |
| ATXN3L |  |  |  |  |
|  | cg07186939 | 3'UTR Open_Sea | 1.621 | 0.0062 |
|  | cg00595697 | TSS1500 Open_Sea | 1.264 | 0.26 |
|  | cg12615934 | TSS1500 Open_Sea | 1.418 | 0.1 |
|  | cg06793890 | 1stExon;5'UTR Open_sea | 1.432 | 0.099 |
| JOSD1 |  |  |  |  |
|  | cg03088955 | 3'UTR S_Shelf | 1.527 | 0.021 |
|  | cg01138530 | TSS1500 Island | 0.67 | 0.026 |
|  | cg11157800 | 1stExon;5'UTR-Island | 1.17 | 0.38 |
|  | cg26162007 | TSS200-Island | 0.881 | 0.47 |
|  | cg09260951 | TSS200-Island | 1.626 | 0.021 |
|  | cg11355601 | TSS200-Island | 0.856 | 0.46 |
|  | cg26380756 | TSS1500-Island | 1.408 | 0.052 |
|  | cg00566492 | TSS1500-Island | 1.363 | 0.13 |
|  | cg09576223 | Body N_Shelf | 1.327 | 0.11 |
|  | cg27610821 | TSS1500-Island | 0.665 | 0.023 |
|  | cg19658332 | TSS1500-S_Shore | 0.612 | 0.014 |
|  | cg20659657 | TSS200-Island | 1.554 | 0.036 |
|  | cg25697769 | TSS1500-Island | 1.828 | 0.004 |
|  | cg06711259 | 1stExon;N_Shore | 0.455 | 3.10E-05 |
|  | cg16722058 | TSS200-Island | 1.109 | 0.56 |
|  | cg18767321 | TSS200-Island | 1.809 | 0.001 |
|  | cg09991975 | TSS1500-Island | 1.594 | 0.017 |
| JOSD2 |  |  |  |  |
|  | cg17168820 | 1stExon;5'UTR-Island | 0.753 | 0.11 |
|  | cg22266470 | TSS1500-Island | 1.433 | 0.053 |
|  | cg13521229 | Body N_Shore | 0.88 | 0.47 |
|  | cg03523122 | 5'UTR-Island | 1.166 | 0.38 |
|  | cg18708810 | Body N_Shelf | 1.735 | 0.012 |
|  | cg07775501 | TSS200-Island | 1.126 | 0.5 |

**Supplement TABLE 3 Correlation of JOSD1 expression with immunomodulators based on TISIDB  database**

| Immunomodulators | JOSD1 expression TISIDB rho, n=373 | *P* |
| --- | --- | --- |
| CCL14 | -0.233 | 5.92e-06 |
| CCL28 | 0.219 | 2.14e-05 |
| CD160 | -0.208 | 5.54e-05 |
| CD56bright | -0.201 | 9.26e-05 |

**Supplement TABLE 4 Correlation of JOSD2 expression with immunomodulators based on TISIDB  database**

| Immunomodulators | JOSD2 expression TISIDB rho, n=373 | *P* |
| --- | --- | --- |
| CD274 | -0.293 | 8.99e-09 |
| KDR | -0.269 | 1.4e-07 |
| LGALS9 | 0.237 | 3.77e-06 |
| PVRL2 | 0.335 | 4.28e-11 |
| TGFBR1 | -0.265 | 2.25e-07 |
| CD28 | -0.205 | 6.84e-05 |
| IL6R | -0.405 | <2.2e-16 |
| TNFRSF4 | 0.304 | 2.53e-09 |
| TNFRSF14 | 0.325 | 1.7e-10 |
| TNFRSF18 | 0.287 | 1.83e-08 |
| TNFRSF25 | 0.259 | 4.3e-07 |
| HLA-A | 0.257 | 5.43e-07 |
| HLA-DMA | 0.218 | 2.33e-05 |
| CCR4 | -0.268 | 1.61e-07 |
