# Supplementary figures and images for: MJDs family members: Potential prognostic targets and immune-associated biomarkers in hepatocellular carcinoma
Source: Front Genet. 2022 Sep 9;13:965805. doi: 10.3389/fgene.2022.965805 (PMC9500549; doi:10.3389/fgene.2022.965805)

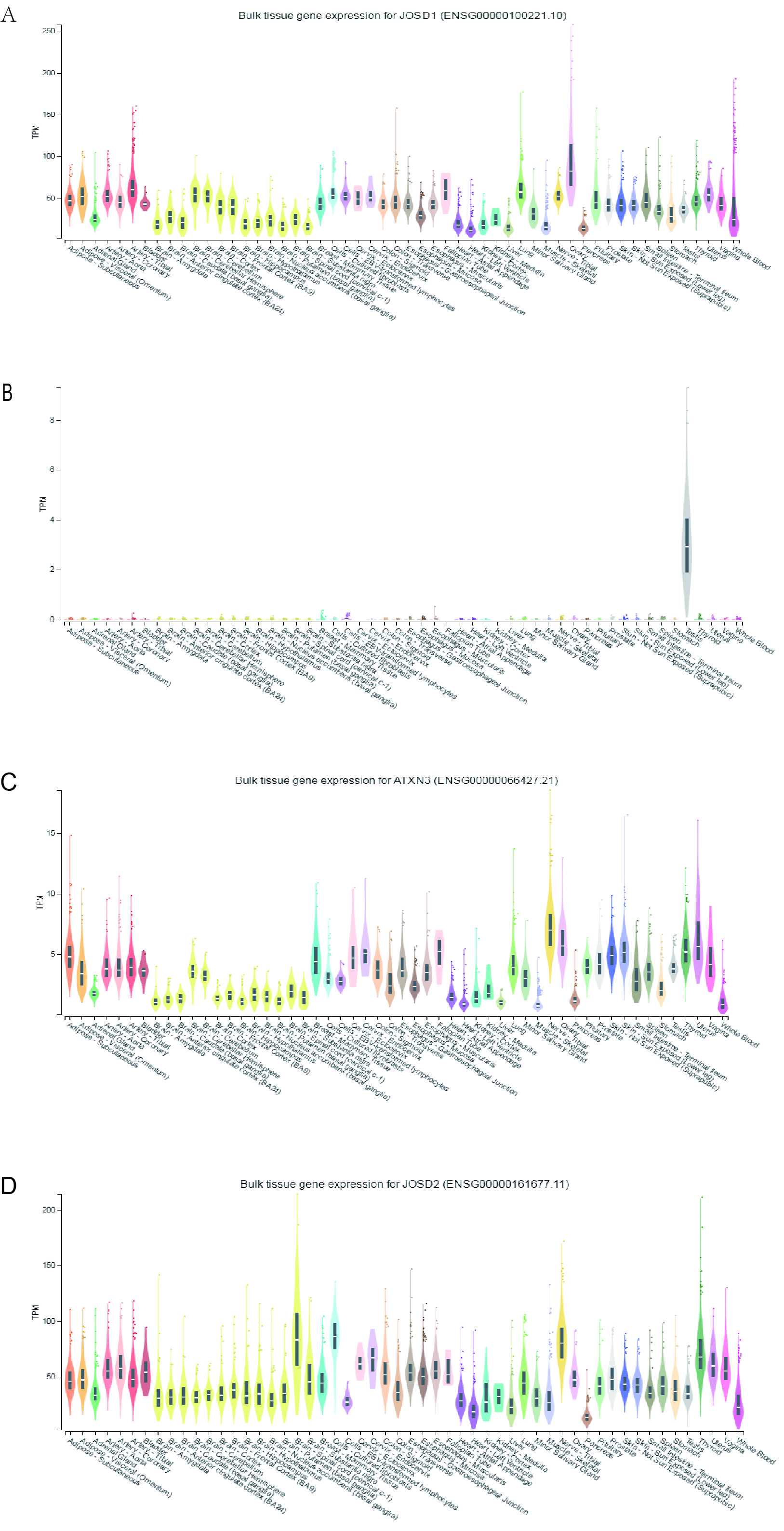

Supplement: Supplementary file 1 [file DataSheet1.ZIP › Data sheet1/supplement figure/supplement figure 1.jpg]

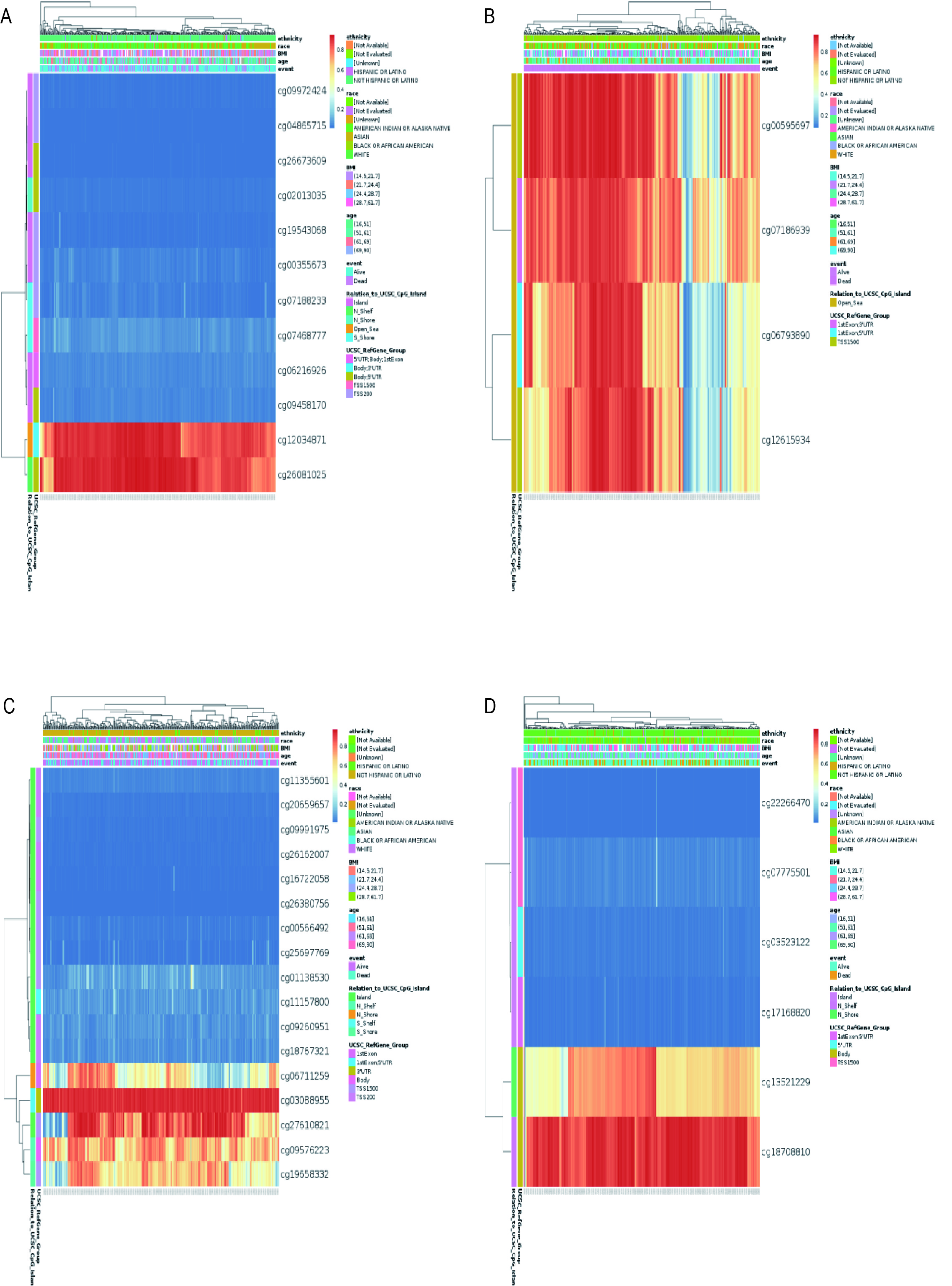

Supplement: Supplementary file 1 [file DataSheet1.ZIP › Data sheet1/supplement figure/supplement figure 2.jpg]

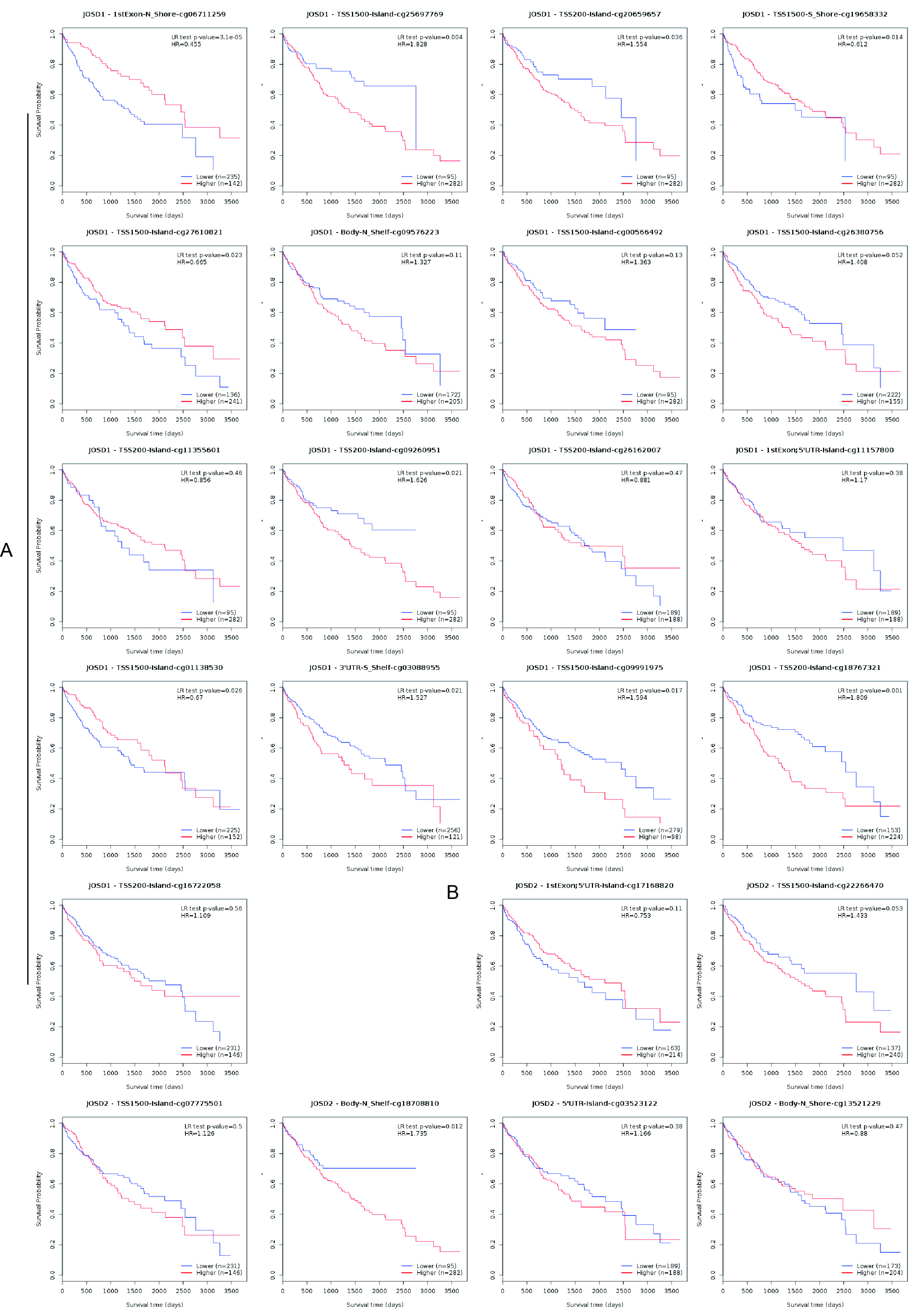

Supplement: Supplementary file 1 [file DataSheet1.ZIP › Data sheet1/supplement figure/supplement figure 3.jpg]

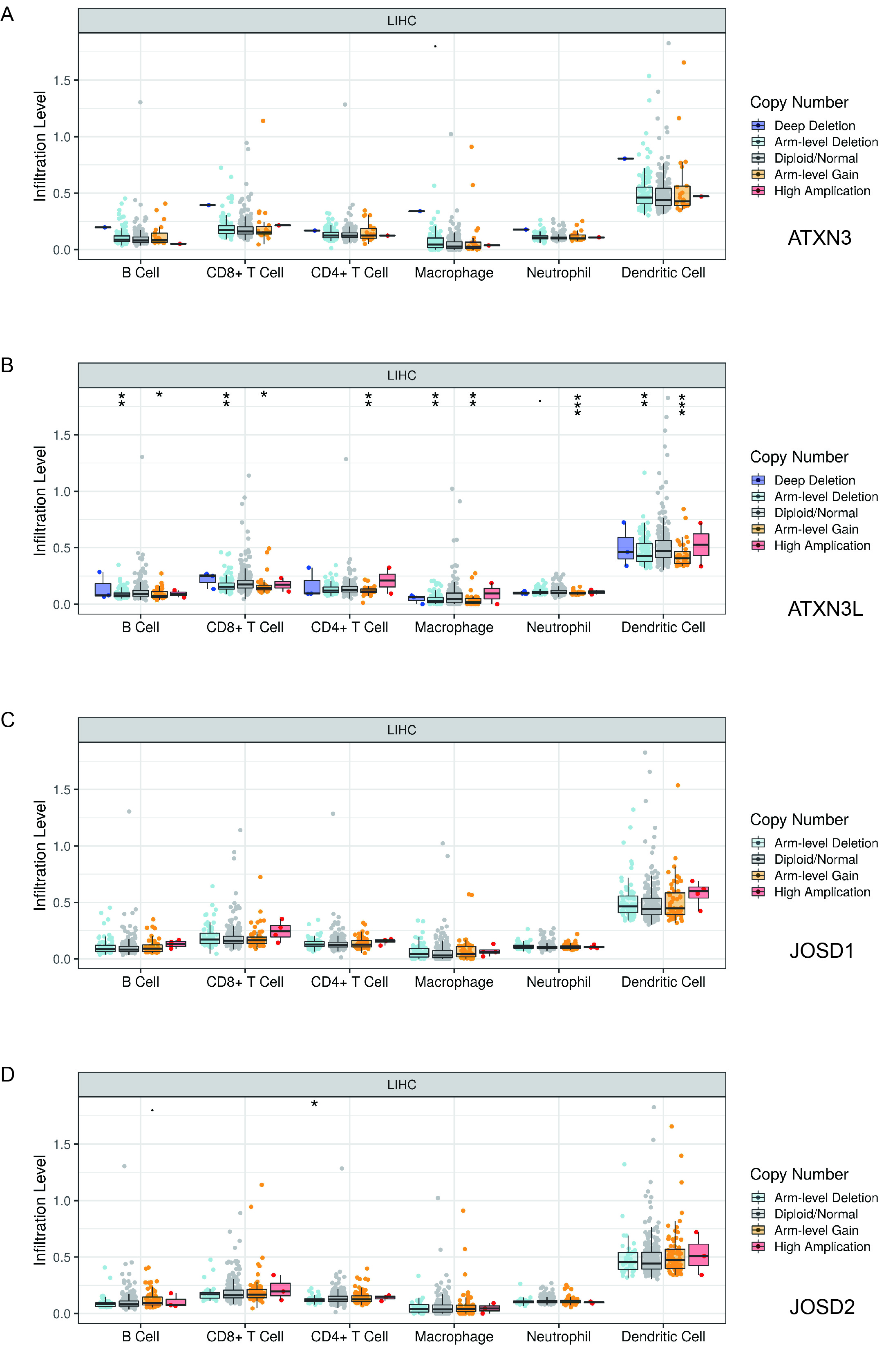

Supplement: Supplementary file 1 [file DataSheet1.ZIP › Data sheet1/supplement figure/supplement figure 4.jpg]

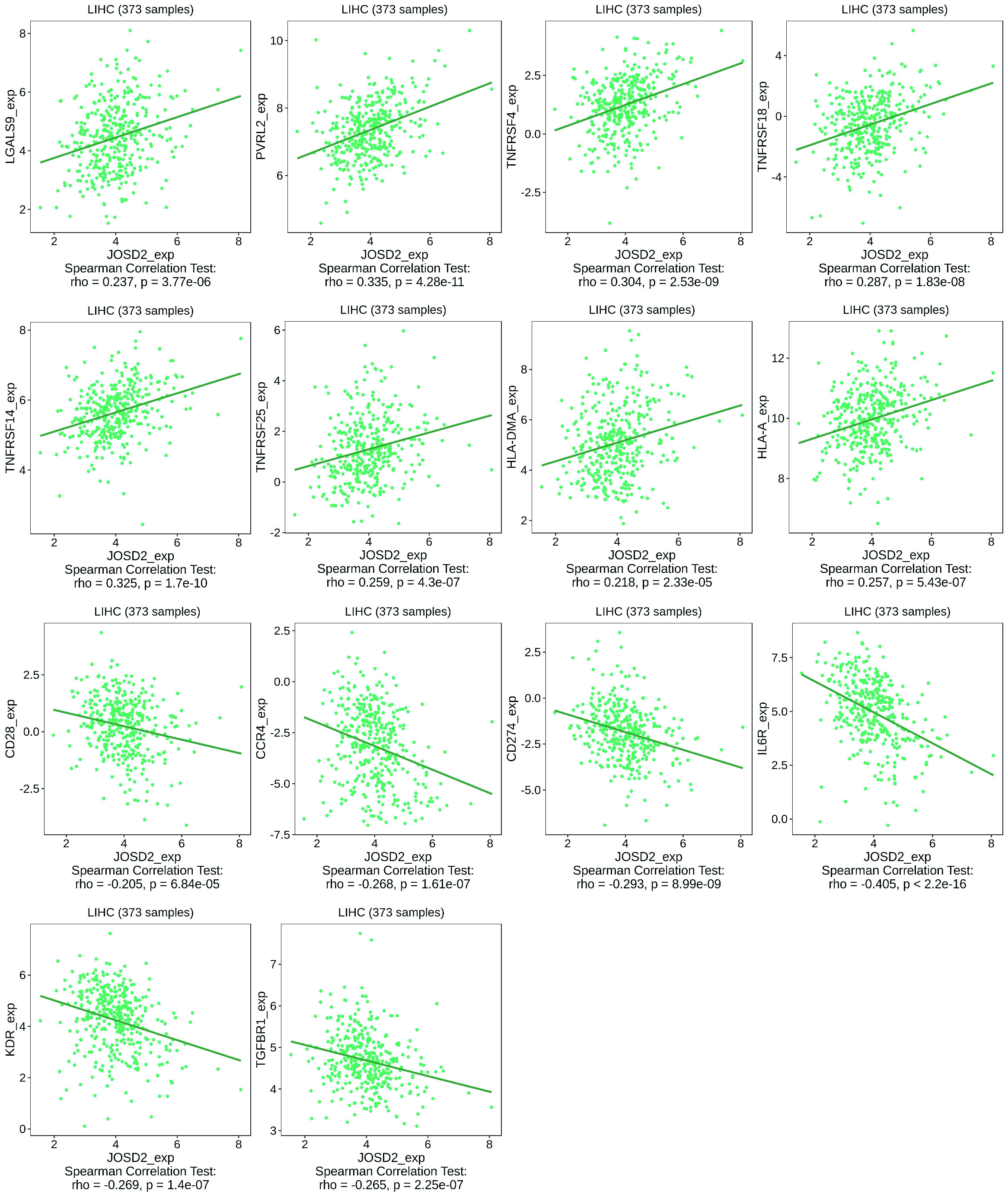

Supplement: Supplementary file 1 [file DataSheet1.ZIP › Data sheet1/supplement figure/supplement figure 5.jpg]

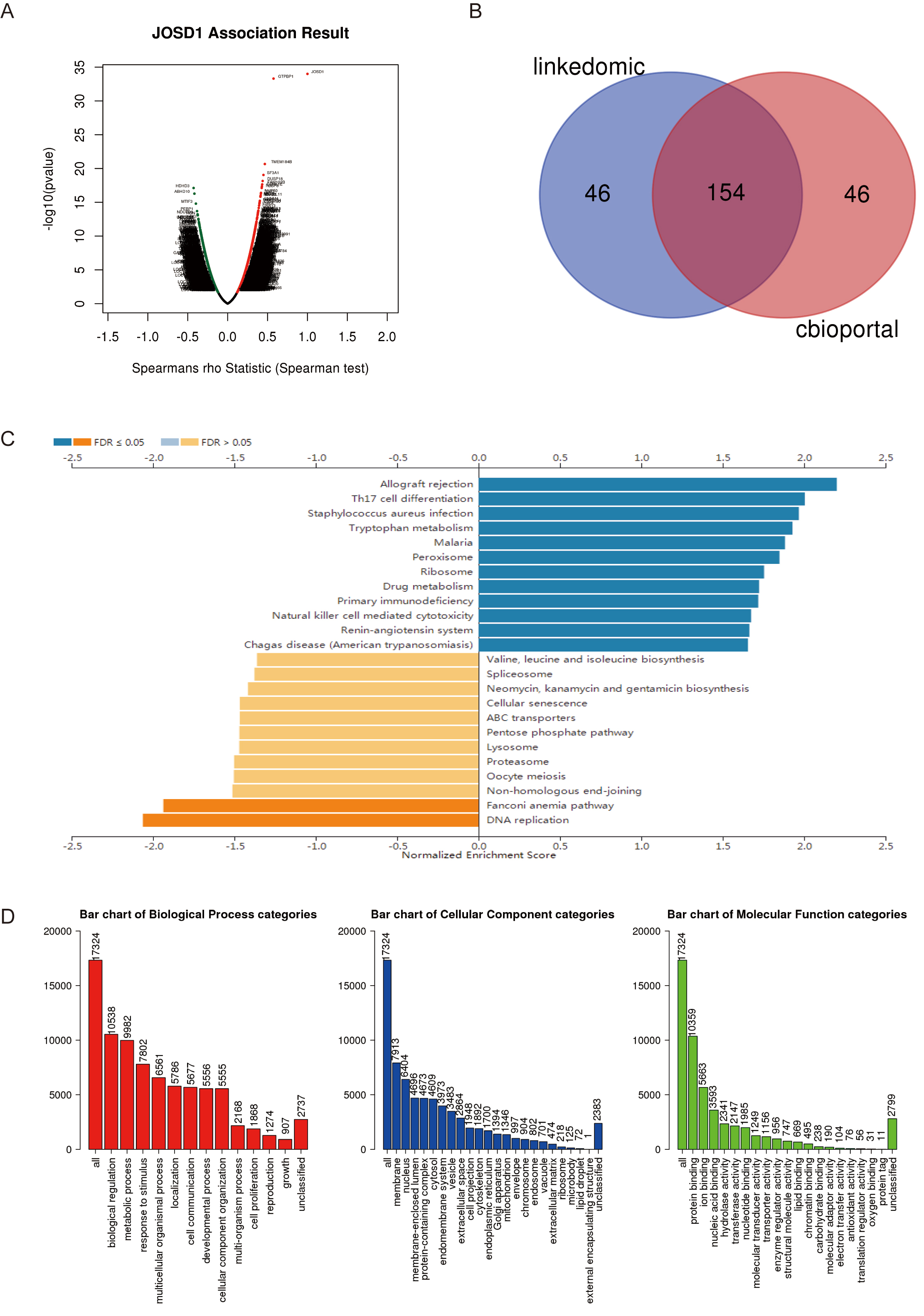

Supplement: Supplementary file 1 [file DataSheet1.ZIP › Data sheet1/supplement figure/supplement figure 6.jpg]

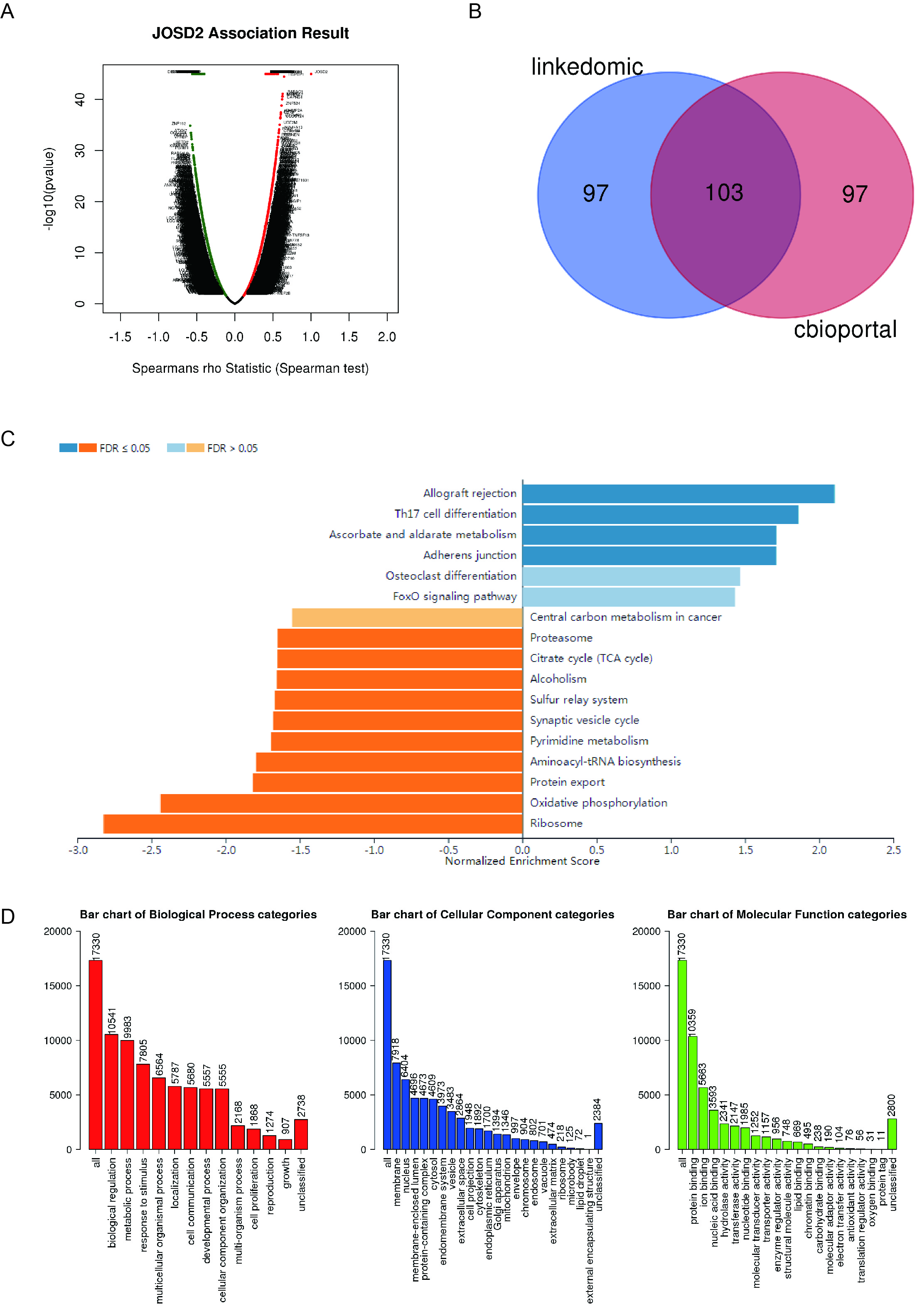

Supplement: Supplementary file 1 [file DataSheet1.ZIP › Data sheet1/supplement figure/supplement figure 7.jpg]

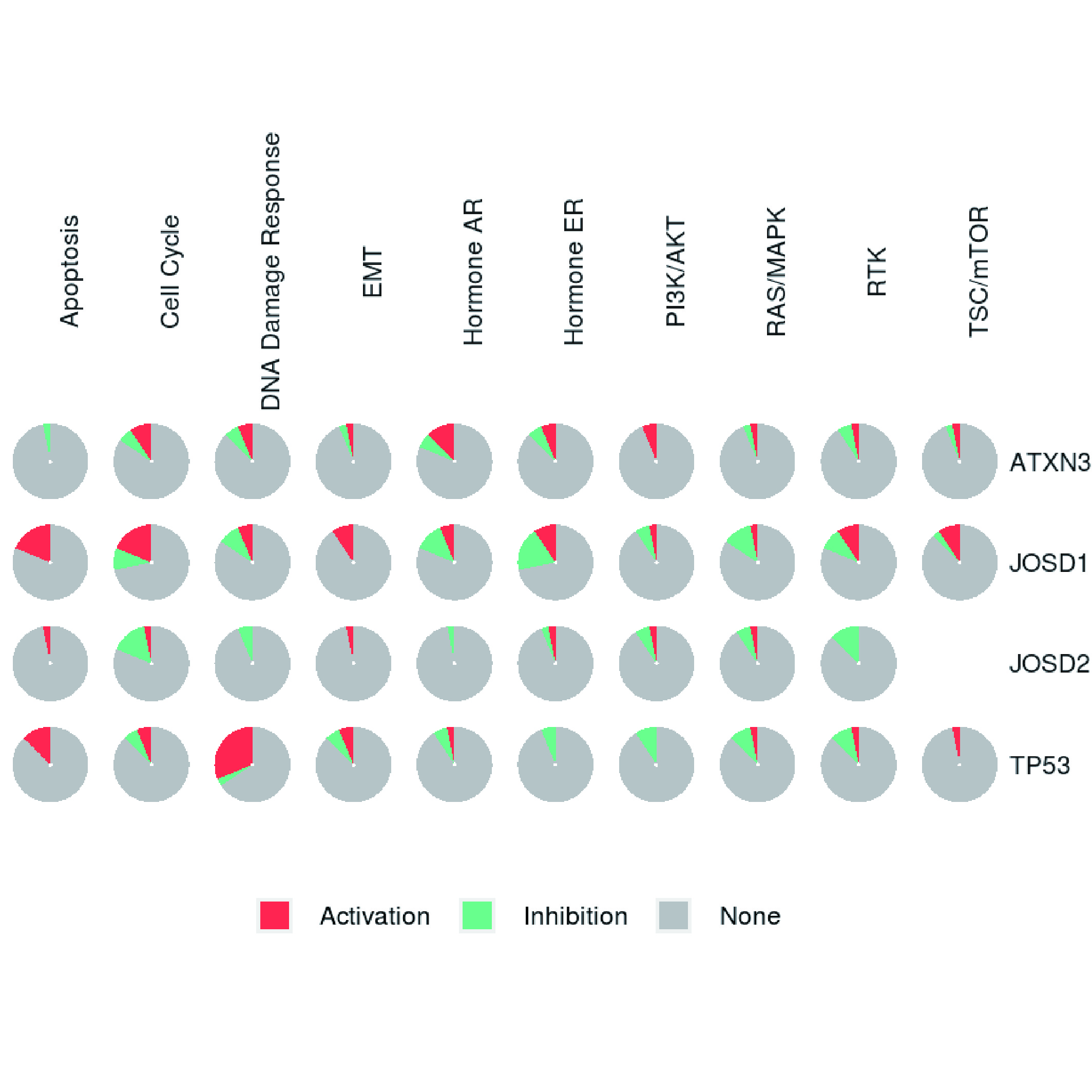

Supplement: Supplementary file 1 [file DataSheet1.ZIP › Data sheet1/supplement figure/supplement figure 8.jpg]
